# Pre-CT risk stratification using the D-dimer/pCO₂ ratio in D-dimer–positive emergency department patients: diagnostic accuracy study

**DOI:** 10.1186/s12873-025-01395-6

**Published:** 2025-11-17

**Authors:** Cem Yıldırım, Ahmet Aykut, Ertuğ Günsoy, Mehmet Veysel Öncül

**Affiliations:** Emergency Department, SBU. Van Education and Research Hospital, Van, Turkey

**Keywords:** Pulmonary embolism, D-dimer, Carbon dioxide, Blood gas analysis, Clinical decision support, Diagnostic accuracy

## Abstract

**Background:**

The diagnostic utility of the D-dimer/pCO₂ ratio for pulmonary embolism (PE) risk stratification has not been fully established. This study evaluated its diagnostic performance among emergency department patients with positive age-adjusted D-dimer results undergoing computed tomography pulmonary angiography (CTPA).

**Methods:**

This retrospective diagnostic accuracy study included 698 adult patients with positive age-adjusted D-dimer results, venous blood gas (VBG) pCO₂ measurements, and definitive CTPA interpretation. The D-dimer/pCO₂ ratio was calculated, and receiver operating characteristic (ROC) analysis was performed. Optimal and exploratory thresholds were assessed for overall PE detection and for excluding central PE. Robustness was tested using bootstrap validation and subgroup AUC comparisons. Decision curve analysis (DCA) was applied to evaluate clinical utility.

**Results:**

PE was confirmed in 90 patients (12.9%). The ratio demonstrated good discrimination (AUC: 0.811, 95% CI: 0.775–0.847). At the optimal cut-off (44.91), sensitivity was 82.2% and specificity 71.1%, with a negative predictive value (NPV) of 96.4%. A lower cut-off (18.1) identified 91 patients with no observed PE (0/91; 95% CI upper bound for false negatives ≈ 4.0%). A higher threshold (61.25) identified 515 patients below this value, among whom no central PE was observed (0/515; 95% CI upper bound ≈ 0.7%). Discriminative ability was preserved across age groups (AUC range: 0.737–0.836). DCA showed modest, range-specific net benefit for incorporating the ratio within a low-to-intermediate threshold band.

**Conclusion:**

In D-dimer–positive ED patients already being considered for CTPA, the D-dimer/pCO₂ ratio is an adjunctive imaging triage indicator rather than a stand-alone test and may help inform the imaging workflow in this defined context. These findings should not be extrapolated to D-dimer–negative patients or those with very high pretest probability.

**Supplementary Information:**

The online version contains supplementary material available at 10.1186/s12873-025-01395-6.

## Introduction

Pulmonary embolism (PE) is a potentially life-threatening condition that often presents with nonspecific symptoms, making timely diagnosis challenging in emergency medicine. D-dimer testing is widely used to rule out PE in low- to intermediate-risk patients because of its high sensitivity. However, its limited specificity—particularly in older or medically complex patients—leads to false positives and potential overuse of computed tomography pulmonary angiography (CTPA) [1, 2].

To reduce unnecessary imaging, prior studies combined D-dimer with physiologic indicators of ventilation–perfusion mismatch, especially measures related to carbon dioxide (CO₂) exchange. Parameters such as alveolar dead-space fraction, end-tidal CO₂ (etCO₂), and the arterial–end-tidal CO₂ gradient have been evaluated alongside D-dimer [3–5]. Collectively, these studies suggest that a normal D-dimer with preserved CO₂ exchange confers excellent negative predictive value (NPV), whereas elevated D-dimer with abnormal CO₂ dynamics increases the likelihood of PE [6–8].

Although carbon dioxide–based metrics such as end-tidal CO₂ (etCO₂) have been investigated alongside D-dimer and clinical probability to assess pulmonary embolism risk, these studies have relied on capnography rather than blood gas analysis [9]. Given the routine use and accessibility of both venous blood gas (VBG) and D-dimer testing in emergency departments, this ratio may offer a simple, physiologically grounded adjunctive imaging triage indicator in suspected PE cases.

We aimed to evaluate whether, in ED adults with positive age-adjusted D-dimer who are already being considered for CTPA, the D-dimer/pCO₂ ratio provides add-on imaging triage value. Specifically, we (i) quantified overall discrimination at a Youden-optimized threshold (44.91), (ii) assessed a failure-rate–anchored rule-out candidate (18.1), (iii) examined a high-risk boundary for central PE (61.25), and (iv) used decision-curve analysis to contextualize clinical utility across relevant threshold probabilities.

## Methods

### Study design and setting

This was a retrospective, single-center diagnostic accuracy study conducted in the emergency department of a tertiary care hospital between January 1, 2023, and December 31, 2024. The study aimed to evaluate the diagnostic performance of the D-dimer/pCO₂ ratio in predicting PE among adult patients who underwent CTPA. D-dimer and venous blood gas (VBG) were obtained concurrently during the initial ED evaluation, and CTPA was performed within 2 h of VBG as part of our institutional diagnostic pathway. The cohort comprised D-dimer–positive emergency department patients who underwent CTPA; patients with very high pretest probability who proceeded directly to CTPA without prior D-dimer were not represented. Accordingly, we evaluated the D-dimer/pCO₂ ratio as an add-on imaging triage aid rather than a stand-alone rule-out test.

**Fig. 1:**
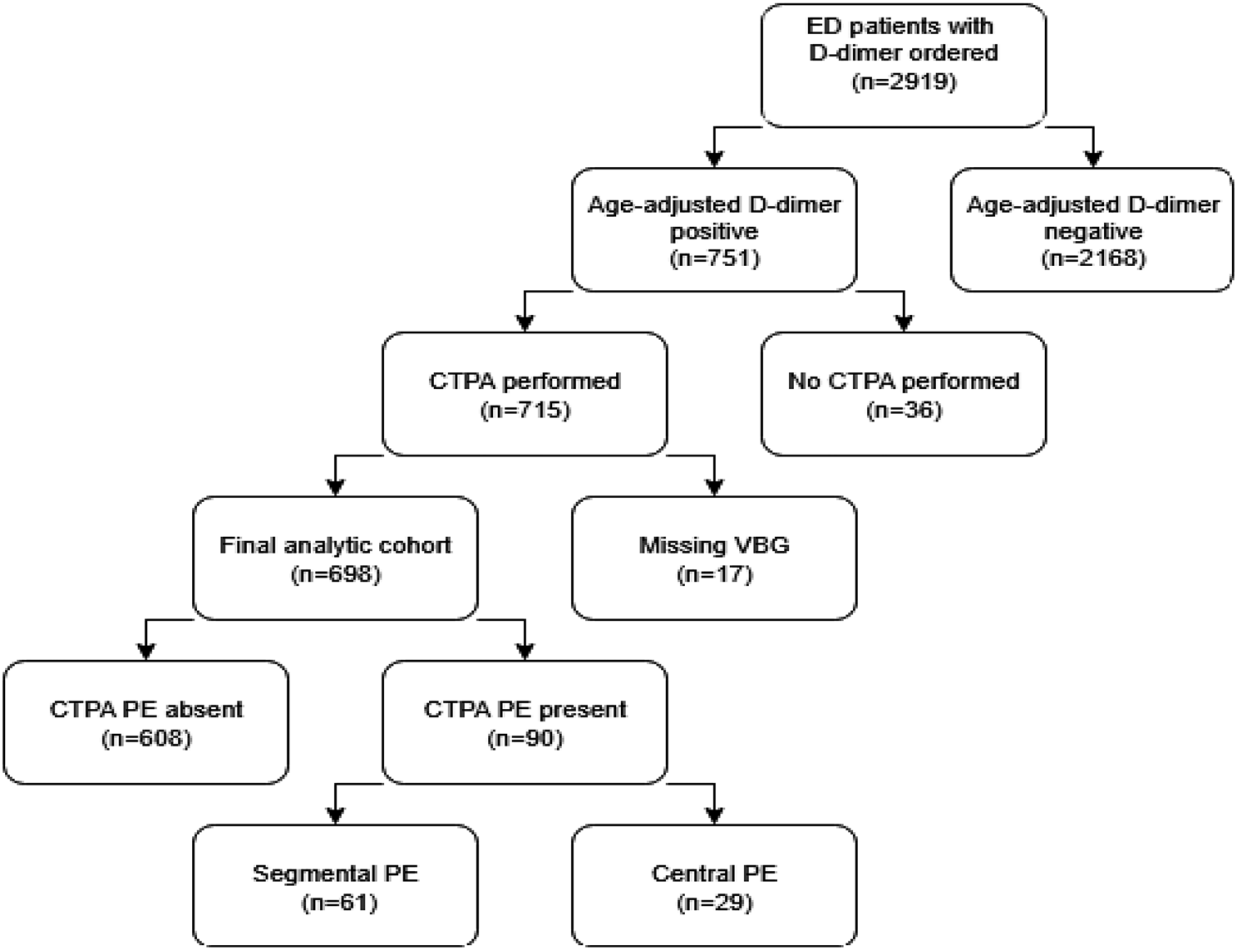
Study flow diagram. Patient selection and exclusions among emergency department patients for whom a D-dimer was ordered. The final analytic cohort included 698 patients with positive age-adjusted D-dimer, complete venous blood gas (VBG), and CTPA results. Downstream boxes display reference-standard outcomes: PE absent (*n* = 608) and PE present (*n* = 90), subdivided into segmental PE (*n* = 61) and central PE (*n* = 29). Note: Patients with high pretest probability who underwent direct CTPA without prior D-dimer testing were not captured by the inclusion pathway

In addition to assessing overall diagnostic performance, we also explored whether specific thresholds of the D-dimer/pCO₂ ratio could support risk stratification, including potential exclusion of PE or central (high clot burden) PE in defined subgroups. The patient selection process and exclusions are summarized in the study flow diagram (Fig. 1).

No a priori sample size calculation was performed due to the retrospective design; the sample size was determined by data availability across the study period.

### Inclusion and exclusion criteria

Adult patients (aged ≥ 18 years) were eligible if they met all of the following:

1. a positive D-dimer result based on age-adjusted thresholds (defined as ≥ 500 ng/mL for patients aged < 50 years, and ≥ age × 10 ng/mL for those aged ≥ 50 years);
2. a VBG sample obtained during initial ED evaluation with a recorded pCO₂ value;
3. a completed CTPA with a definitive radiological interpretation regarding the presence or absence of PE.

Patients were excluded if they had a negative age-adjusted D-dimer result, lacked complete VBG or D-dimer data, or did not undergo CTPA or had a non-diagnostic interpretation. These criteria ensured that the study cohort represented patients for whom both laboratory and imaging data were available to allow reliable assessment of the diagnostic performance of the D-dimer/pCO₂ ratio.

Patient inclusion and exclusion counts (D-dimer–negative, missing VBG, no CTPA, non-diagnostic CTPA) are detailed in the study flow diagram (Fig. 1) and are consistent with Table 1 totals. Missing data were handled by complete-case analysis; no imputation was performed.

**Table 1:** Patient characteristics of the study cohort (*n* = 698)

Variable	Overall (*n* = 698)	No PE (*n* = 608)	Segmental PE (*n* = 61)	Central PE (*n* = 29)
Age, years (mean ± SD)	60.1 ± 18.8	59.2 ± 18.9	63.0 ± 17.2	72.5 ± 15.2
Male, n (%)	260 (37.2)	224 (36.8)	24 (39.3)	12 (41.4)
D-dimer, ng/mL (median [IQR])	1390 [944–2511]	1258 [914–2094]	2875 [1626–5060]	8996 [5228–24756]
pCO₂, mmHg (median [IQR])	41.0 [36.5–46.1]	41.3 [36.68–46.30]	38.9 [35.2–43.9]	40.4 [35.7–47.1]
pH (median [IQR])	7.39 [7.37–7.42]	7.39 [7.37–7.42]	7.40 [7.37–7.43]	7.37 [7.32–7.41]
Lactate, mmol/L (median [IQR])	1.80 [1.40–2.40]	1.70 [1.40–2.30]	2.00 [1.60–2.70]	2.70 [1.80–3.90]
D-dimer/pCO₂ ratio (median [IQR])	33.6 [22.8–64.0]	30.7 [21.6–50.0]	75.3 [42.6–116.6]	262.2 [140.7–490.7]

Data are presented as n (%) for categorical variables and summary statistics for continuous variables (Age as mean ± SD; other continuous measures as median [IQR], unless otherwise specified). pCO₂ values are from venous blood gas (mmHg). Central PE denotes embolism involving the main pulmonary artery or both right and left pulmonary arteries. Percentages are calculated within the stated column denominators

### Variables and definitions

The D-dimer/pCO₂ ratio was calculated by dividing the D-dimer concentration (ng/mL) by the pCO₂ value (mmHg) obtained from a VBG sample drawn during the initial emergency department evaluation.

PE was considered present if the CTPA report confirmed thromboembolic involvement in any pulmonary arterial segment. For the purposes of exploratory subgroup analysis, PE was further categorized as:

- Central PE (high clot burden): defined as embolism involving the main pulmonary artery or both right and left pulmonary arteries, irrespective of hemodynamic status.
- Non-central PE: defined as embolism confined to lobar, segmental, or subsegmental branches.

### Data collection and outcomes

Data were retrospectively collected from the hospital’s electronic health record system, laboratory information system, and the radiology picture archiving and communication system (PACS). For each patient meeting the inclusion criteria, demographic characteristics (age, sex), clinical presentation, and laboratory parameters (D-dimer level, venous blood gas results including pCO₂) were extracted. Radiology reports were reviewed to confirm the presence, absence, or type of pulmonary embolism (PE).

pCO₂ values were obtained from venous blood gas (VBG) at presentation. VBG is less invasive, easier to obtain, and routinely performed in emergency settings. Recent ED data demonstrate acceptable agreement between venous and arterial pCO₂ and support venous-to-arterial conversion models; pooled estimates indicate venous pCO₂ is, on average, ~ 4–5 mmHg higher [10, 11]. A meta-analysis reported a mean difference of 4.4–5.3 mmHg, supporting its clinical utility in hemodynamically stable patients [12]. While arterial blood gas (ABG) analysis remains essential for assessing oxygenation (PaO₂), ED studies indicate that VBG provides clinically useful estimates of pCO₂, pH, and bicarbonate for most patients, with acceptable V–A agreement supported by conversion models [10, 12]. Accordingly, venous pCO₂ was considered a clinically acceptable surrogate for use in our ratio-based analysis.

All CTPA examinations were interpreted by a single radiology specialist in routine clinical practice. Specific years of reader experience were not systematically recorded. Reports were retrieved directly from the radiology PACS system without re-interpretation for this study, thereby minimizing investigator bias.

The primary outcome was the diagnostic accuracy of the D-dimer/pCO₂ ratio for detecting PE as confirmed by CTPA.

Secondary outcomes included:

- The ability of exploratory, cohort-derived thresholds (18.1 and 61.25) to either exclude any PE (rule-out candidate) or identify central PE (high-risk flag),
- Subgroup diagnostic performance by age categories, and.
- The potential clinical utility of incorporating the ratio into imaging strategies as assessed by decision curve analysis.

Data completeness was verified prior to analysis. Patients with missing essential variables (D-dimer, pCO₂, or CTPA interpretation) were excluded, as detailed in the flow diagram (Fig. 1).

### Statistical analysis

All analyses focused on the independent diagnostic performance of the D-dimer/pCO₂ ratio in D-dimer–positive ED patients already being considered for CTPA; combined strategies with validated clinical decision rules (e.g., Wells, YEARS) were not evaluated. Receiver operating characteristic (ROC) analysis was used to assess discrimination, with area under the curve (AUC) and 95% confidence intervals. Two exploratory, cohort-derived thresholds were also evaluated: 18.1 as a rule-out candidate (associated with NPV 100% in this cohort) and 61.25 as a high-risk flag for central PE; by contrast, the discrimination-oriented cut-off was selected by Youden’s index (44.91).

The acceptable failure rate (%) was defined a priori using the ISTH framework as 1.82 + (0.00528 × the cohort prevalence in %); with a prevalence of 12.9%, this corresponds to approximately 1.9% (15). To quantify precision and compare strata, we applied bootstrap resampling (1,000 iterations) for AUC confidence intervals and used the DeLong method for pairwise AUC comparisons across predefined subgroups. Standard accuracy metrics (sensitivity, specificity, predictive values, and likelihood ratios) were calculated with 95% confidence intervals for key thresholds. Age-stratified performance was summarized across five groups (< 50, 50–59, 60–69, 70–79, ≥ 80 years) by reporting AUCs (with 95% CIs) within each stratum.

Decision curve analysis (DCA) was performed to evaluate clinical net benefit across threshold probabilities from 0% to 50%, comparing the ratio-guided strategy (index test at ratio ≥ 44.91) with the default treat-all and treat-none approaches. Net benefit was calculated as NB = (TP/N) − (FP/N) × (pt/(1 − pt)); treat-none is identically zero, and the treat-all curve crosses NB = 0 at the threshold probability equal to the study prevalence (pt = 0.129). For decision-level interpretability, the expected CTPA reduction per 100 tested was calculated as (number meeting the rule-out criterion / total) × 100.

### Software

Statistical analyses and data processing were conducted using Python version 3.11, with the scikit-learn and pandas libraries.

## Results

### Patient characteristics

A total of 698 patients with positive age-adjusted D-dimer results and complete diagnostic data were included in the analysis. The mean age was 60.1 ± 18.8 years, and 37.2% (*n* = 260) were male. PE was diagnosed in 90 patients (12.9%) based on CTPA findings. The median D-dimer level was 1390 ng/mL (interquartile range: 944–2511), and the mean venous pCO₂ was 42.1 ± 8.9 mmHg, as shown in Table 1.

### ROC analysis and optimal cut-off

**Fig. 2:**
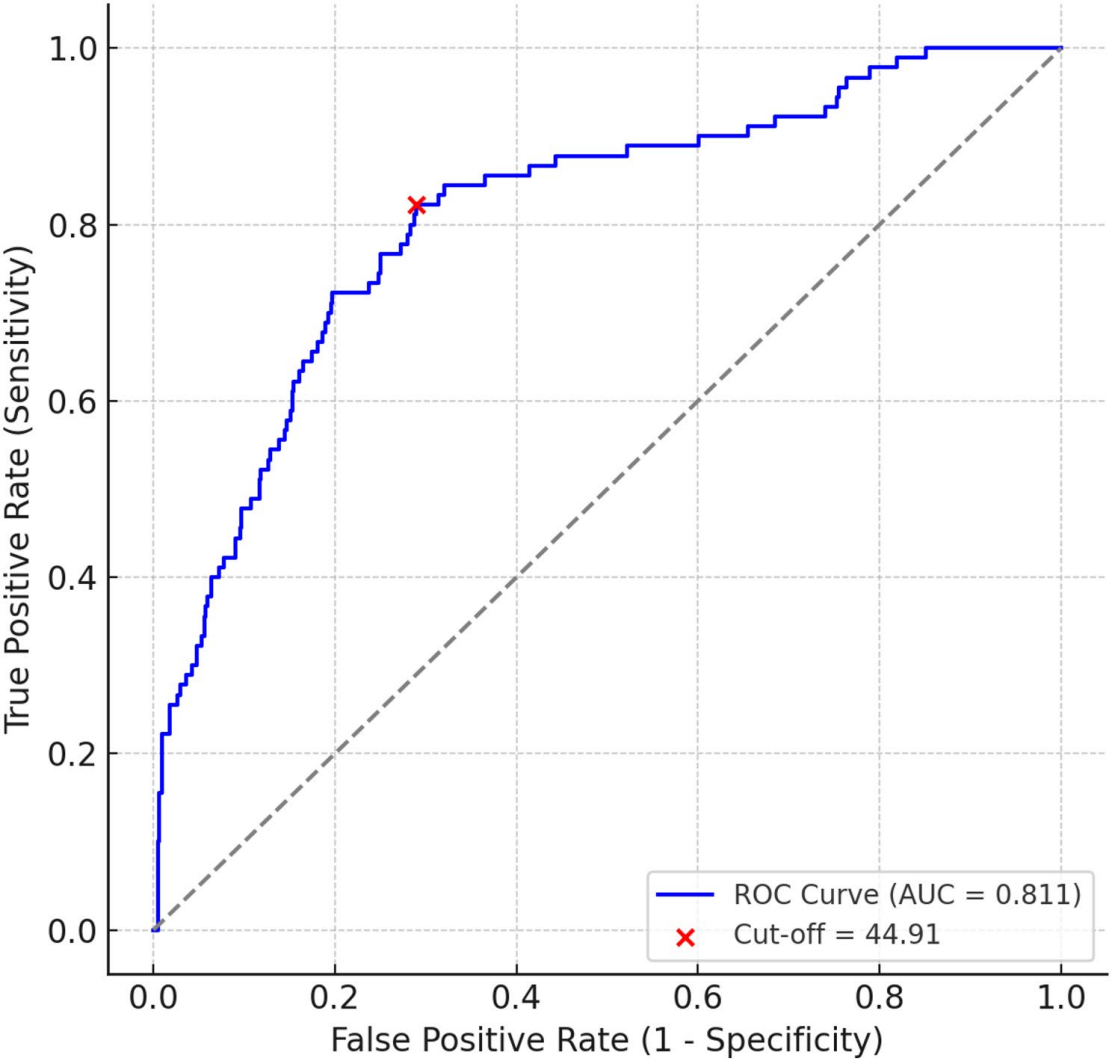
ROC curve for D-dimer/pCO₂ ratio. Receiver operating characteristic (ROC) curve demonstrating diagnostic performance for pulmonary embolism (PE). The optimal cut-off (44.91) is indicated (AUC = 0.811)

The D-dimer/pCO₂ ratio demonstrated good diagnostic performance in distinguishing PE-positive from PE-negative patients, with an area under the ROC curve (AUC) of 0.811 (95% CI: 0.775–0.847; Fig. 2, Supplementary Table 1). The optimal cut-off value identified using Youden’s index was 44.91. The corresponding 2 × 2 contingency data are shown in Table 2, and the detailed performance metrics—including sensitivity (82.2%), specificity (71.1%), and negative predictive value (96.43%)—are presented in Table 3.

**Table 2:** Diagnostic contingency tables for three clinically relevant D-dimer/pCO₂ thresholds

PE D-dimer/pCO₂ ratio								Central PE D-dimer/pCO₂ ratio			
Cut-off	PE(+)	PE(-)	Total	Cut-off	PE(+)	PE(-)	Total	Cut-off	PE(+)	PE(-)	Total
**≥ 18.1**	90	517	607	**≥ 44.91**	74	176	250	**≥ 61.25**	29	154	183
**< 18.1**	0	91	91	**< 44.91**	16	432	448	**< 61.25**	0	515	515
Total	90	608			90	608			29	669	

At a cut-off of < 18.1, 91 patients tested negative and none had PE, resulting in a sensitivity and negative predictive value (NPV) of 100%. At the optimal diagnostic threshold of ≥ 44.91, 250 patients tested positive, correctly identifying 82.2% of PE cases while yielding 176 false positives. For central PE exclusion, 515 patients had ratios < 61.25, and none were diagnosed with central PE, suggesting this threshold may be useful for safely ruling out high clot burden

**Table 3:** Diagnostic performance metrics for the D-dimer/pCO₂ ratio at three clinically relevant thresholds

Cut-off D-dimer/pCO₂ ratio	PE						Central PE		
	< 18.1			≥ 44.91			< 61.25		
Measure	Value	95% CI		Value	95% CI		Value	95% CI	
		Lower	Upper		Lower	Upper		Lower	Upper
Sensitivity (%)	100	95.98	100	82.2	72.74	89.48	100	88.06	100
Specificity (%)	14.6	12.23	18.05	71.1	67.27	74.63	76.98	73.6	80.12
Positive Predictive Value (%)	14.83	14.41	15.25	29.6	26.43	32.98	15.85	14.09	17.78
Negative Predictive Value (%)	100	96.1	100	96.43	94.52	97.69	100	99.3	100
Positive Likelihood Ratio	1.18	1.14	1.22	2.84	2.43	3.32	4.34	3.78	4.99
Negative Likelihood Ratio	0	N/A	N/A	0.25	0.16	0.39	0	N/A	N/A

The < 18.1 threshold demonstrated 100% sensitivity and NPV, suggesting that patients below this ratio may represent a physiologically low-risk subgroup with minimal likelihood of PE. The ≥ 44.91 cut-off provided a balanced diagnostic profile (82.2% sensitivity, 71.1% specificity, 96.4% NPV) and may aid clinicians in stratifying PE risk among D-dimer–positive patients. At the < 61.25 threshold, no central PE cases were observed, with a sensitivity and NPV of 100%, indicating that this cut-off may offer clinical reassurance regarding the absence of high clot burden in certain patients

For cut-offs with no observed events, likelihood ratios could not be calculated and are reported as N/A. However, for transparency, the binomial 95% confidence intervals for the miss rate are provided

At the 18.1 cut-off, no PE cases were observed (0/91); the upper bound of the 95% CI for the miss rate was ≈ 4.0%

At the 61.25 cut-off, no central PE cases occurred (0/515); the 95% CI upper bound was ≈ 0.7%

To further assess the robustness of these findings, supplementary analyses were performed. Bootstrap validation confirmed the stability of the AUC estimates (Supplementary Table 1). Pairwise AUC comparisons across predefined subgroups are presented in Supplementary Table 2, showing no statistically significant differences.

### Diagnostic accuracy at key cut-offs

#### Cut-off = 44.91 for predicting PE

At the optimal threshold of 44.91, 74 of the 90 PE-positive patients tested above the cut-off (true positives), while 176 PE-negative patients also tested positive (false positives). Conversely, 16 PE-positive patients had ratios below the threshold (false negatives), and 432 PE-negative patients tested negative (true negatives) (Table 2).

This yielded a sensitivity of 82.2%, specificity of 71.1%, PPV of 29.6%, NPV of 96.4%, LR⁺ of 2.84, and LR⁻ of 0.25 (Table 3).

#### Cut-off = 18.1 for supporting PE exclusion

Using a lower threshold of 18.1, 91 patients had ratios below this value, and none were diagnosed with PE (0/91). This corresponded to a sensitivity and NPV of 100.0%. Based on the Clopper–Pearson method, the upper bound of the 95% confidence interval for the false-negative rate was approximately 4.0%, indicating that the risk of missed PE cannot be entirely excluded. Specificity (14.6%) and PPV (14.8%) were low (Tables 2 and 3). These findings suggest that values below 18.1 may help identify patients with very low probability of PE, particularly in settings where imaging is limited or delayed. At the failure-rate–anchored threshold of 18.1, there were 0/91 missed events (0%), corresponding to approximately 13 CTPA avoided per 100 D-dimer–positive patients (see Supplementary File 1).

#### Cut-off = 61.25 for excluding central PE

To explore exclusion of central PE, a threshold of 61.25 was assessed. None of the 515 patients with ratios below this cut-off had central PE (0/515), corresponding to a sensitivity and NPV of 100.0%. The 95% confidence interval for the false-negative rate extended up to 0.7% (Clopper–Pearson), suggesting that the probability of missed central PE is very low but not absolutely zero. Specificity was 76.98% and PPV 15.85% (Tables 2 and 3). Thus, values below 61.25 may help safely exclude central PE, although external validation is required. At 61.25, no central PE occurred below this value (0/29), supporting its use as a high-risk boundary (see Supplementary File 2).

#### Clinical utility: decision curve analysis

**Fig. 3:**
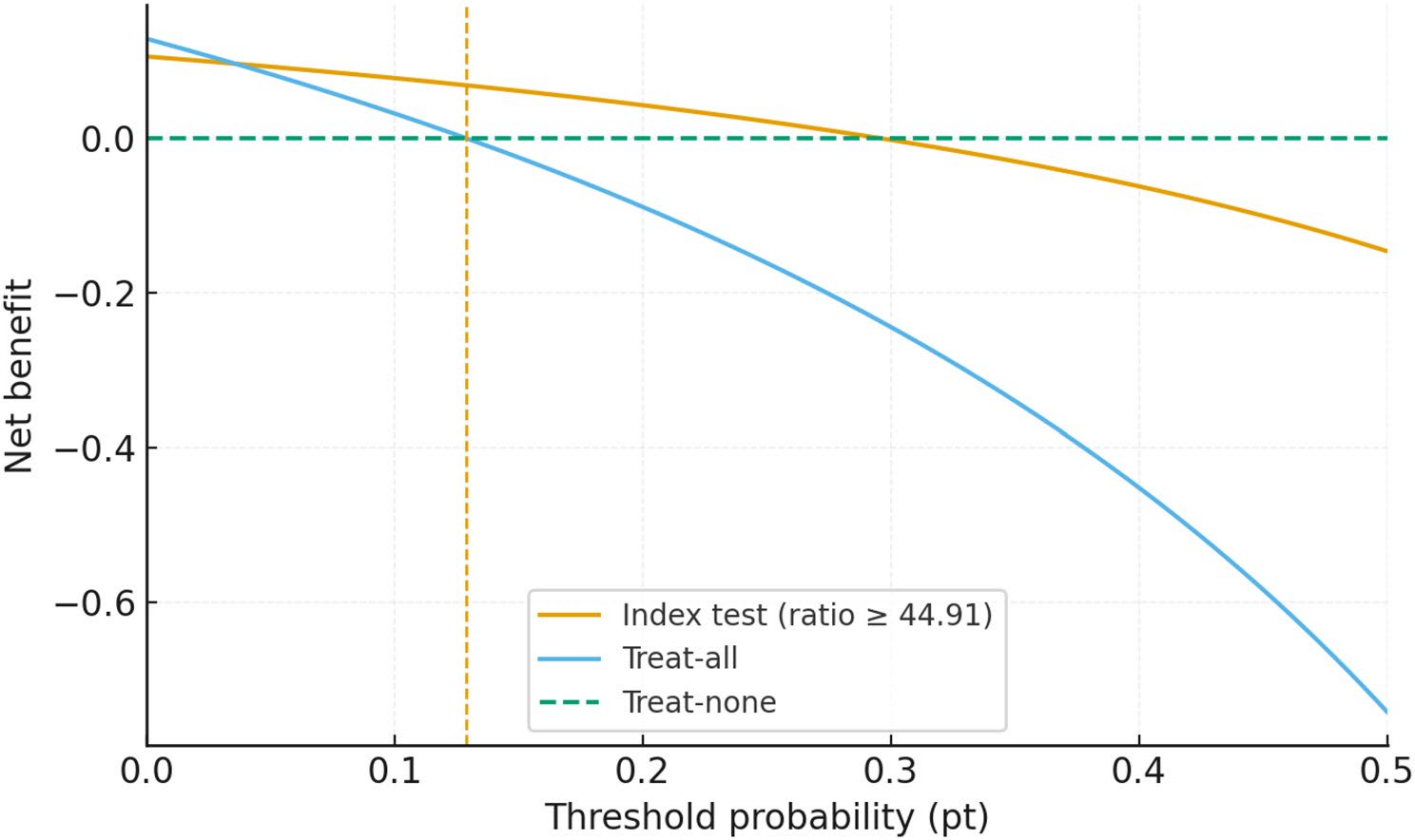
Decision curve analysis net benefit (NB) was calculated as NB = (TP/N) − (FP/N) × (pt/(1 − pt)). Treat-none is identically zero; the treat-all curve crosses NB = 0 at pt = prevalence = 0.129 (vertical guide). For the index test (ratio ≥ 44.91), the zero-crossing occurs at pt = TP/(TP + FP) ≈ 0.30. Reported differences are range-specific and modest and should not be interpreted as dominance across the entire threshold axis

Decision curve analysis (Fig. 3) showed that the treat-all curve intersects the zero-benefit line at the cohort prevalence (pt = 0.129; 12.9%). The treat-none strategy yields zero net benefit across thresholds. For the index test at ratio ≥ 44.91, the net-benefit curve crosses zero at pt ≈ 0.30, beyond which its net benefit becomes negative. Relative to treat-all and treat-none, the index test (ratio ≥ 44.91) offers modest, range-bound gains in net benefit within a limited low-to-intermediate threshold window; beyond that window, differences are minimal and no consistent superiority is claimed.

#### Diagnostic accuracy by age group

**Fig. 4:**
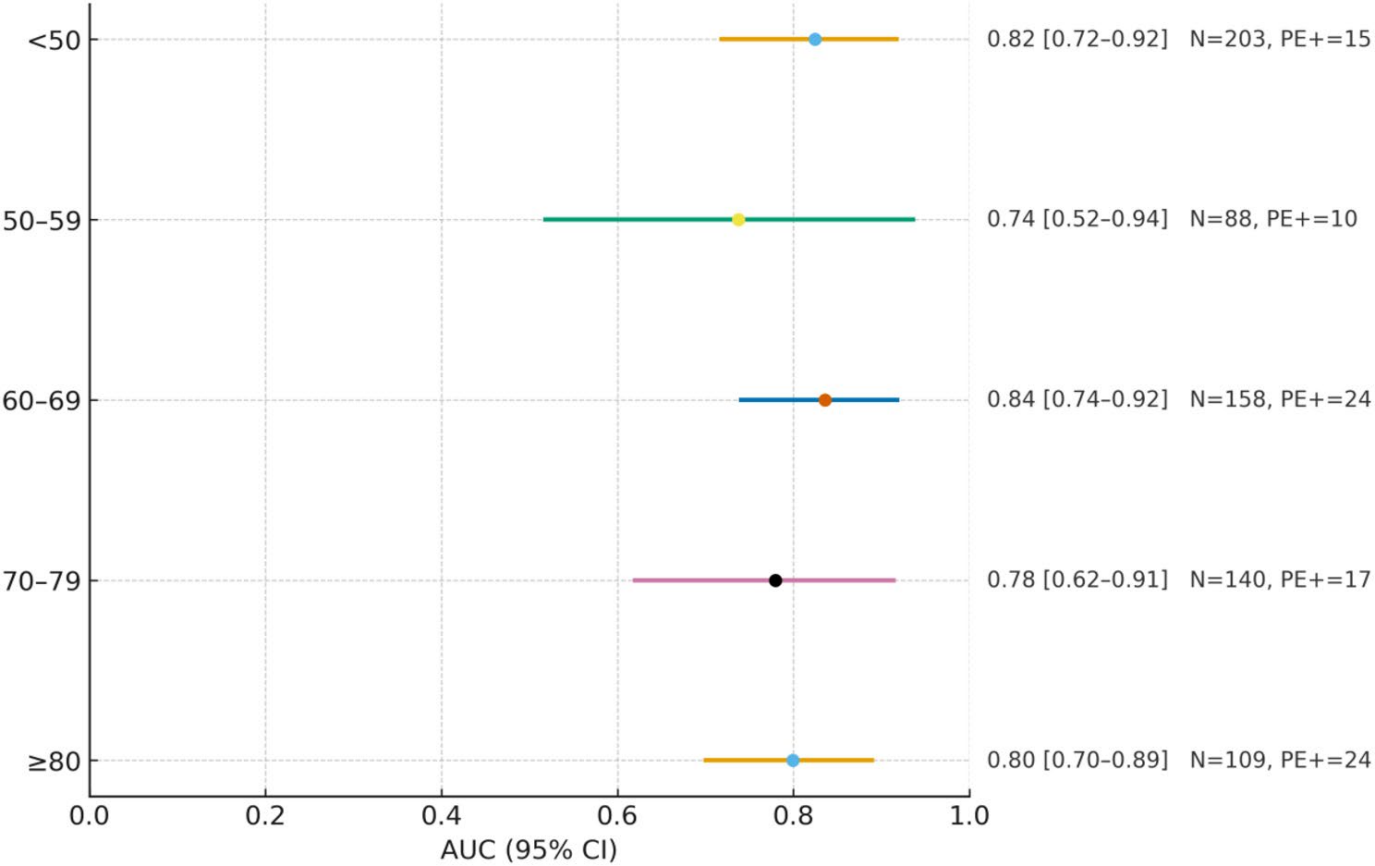
AUC by age group for the D-dimer/pCO₂ ratio. Rows show age strata (< 50, 50–59, 60–69, 70–79, ≥ 80 years). For each stratum, the sample size (N), the number of PE-positive cases, and the area under the ROC curve (AUC) with its 95% confidence interval (CI) are displayed; values are printed next to each row

ROC analyses across five predefined age strata (< 50, 50–59, 60–69, 70–79, ≥ 80) showed AUCs spanning ~ 0.74–0.84, with overlapping 95% confidence intervals across strata (Fig. 4). We therefore do not emphasize a ‘highest’ subgroup; the data suggest that the discriminative ability of the D-dimer/pCO₂ ratio is generally preserved across age groups. Exact AUCs with 95% CIs and per-stratum sample sizes are provided alongside Fig. 4.

## Discussion

PE evaluation in the emergency department requires a probability-guided pathway that balances safety and resource use. D-dimer is indispensable but nonspecific—particularly in older or medically complex patients—driving liberal CTPA use [1, 2]. To refine precision, prior work combined D-dimer with CO₂-based physiologic measures (AVDSf, etCO₂, Pa–EtCO₂) and showed that preserved gas exchange with a negative D-dimer can virtually exclude PE in selected settings; however, most approaches required capnography or arterial sampling, limiting routine uptake [3–5, 7–13].

This study addresses that feasibility gap by operationalizing a simple venous pCO₂–based ratio. Evidence from ED cohorts shows close VBG–ABG pCO₂ agreement and supports practical venous-to-arterial conversion models (typical mean difference ~ 4–5 mmHg), which increases real-world practicality [10–12]. Within this framework, the D-dimer/pCO₂ ratio showed good overall discrimination for identifying PE as an add-on triage aid rather than a stand-alone rule-out or prognostic tool.

We summarize separation with the Youden cut-off (44.91) and then translate performance into decision-oriented thresholds. Anchoring this threshold to the ISTH ‘acceptable failure-rate’ framework (1.82 + 0.00528 × prevalence) yields a target ≈ 1.9% for our cohort (prevalence 12.9%); operationally, applying 18.1 would be expected to avert ~ 13 CTPA per 100 D-dimer–positive patients [14]. This lower boundary is intended as a bedside triage signal compatible with safety-first stewardship, not as a formal stand-alone rule-out tool.

At the upper end of risk, the ratio also helped concentrate concern for large clot burden. Notably, no central PE occurred below 61.25 (0/29), supporting its use as a high-risk boundary to prioritize imaging rather than as a definitive rule-in threshold. Pragmatically, this can assist scanner prioritization when resources are constrained, while still requiring clinical context and external validation.

Decision curve analysis provided supportive context for these thresholds. When contrasted with default strategies, the ratio-based approach offered modest, range-specific gains in net benefit within a clinically meaningful band of threshold probabilities; outside that band, performance was comparable to a default strategy. Accordingly, we present DCA not as stand-alone evidence of broad superiority but as analysis that supports the failure-rate–anchored framework (18.1 and 61.25) and its per-100 decision impact. This pattern aligns with prior observations that incremental tests rarely dominate across the entire threshold spectrum but can add value in well-defined decision bands [3–5, 7–15].

Clinically, the ratio is a decision-refinement aid: values < 18.1 delineate a very-low-risk signal with tangible imaging reduction per 100 D-dimer–positive patients; values ≥ 61.25 flag a high-risk boundary to prioritize imaging and monitoring; and 44.91 remains a separability summary rather than an operating threshold. These inferences should be integrated with clinical probability assessment and local protocols, not used in isolation.

Overall, the ratio appears to be a modest, usable aid rather than a sweeping solution. Prospective, real-world evaluation embedded in routine workflows will show exactly where it brings clarity, reduces low-yield imaging, and helps deliver safer, more timely care.

### Limitations

This study has several limitations. First, its retrospective, single-center design limits external validity and may reduce estimate precision and generalizability across institutions and care settings.

Second, the cohort was restricted to D-dimer–positive patients who underwent CTPA; D-dimer–negative patients and those discharged without imaging were not included. High-pretest-probability cases taken directly to CTPA without prior D-dimer were also not represented. Accordingly, these findings should not be generalized to such presentations, and the D-dimer/pCO₂ ratio should be interpreted as an adjunctive imaging triage aid only in D-dimer–positive patients already being considered for CTPA.

Third, the use of venous rather than arterial pCO₂ may reduce physiologic specificity; however, venous measurements are commonly used and more readily obtainable in routine emergency care.

Fourth, as CT interpretation was performed by a single radiology specialist, inter-reader agreement could not be assessed, and detailed experience metrics for that specialist were unavailable.

Fifth, we did not test the ratio within established rule-based diagnostic pathways (e.g., Wells, YEARS). Therefore, our findings should be regarded as hypothesis-generating until prospectively validated in combination with clinical decision rules.

Finally, the identified cut-off value of 44.91 was derived through retrospective ROC analysis and should be prospectively validated in larger, multicenter cohorts. The additional thresholds of 18.1 and 61.25, used in exploratory analyses for low-risk and central PE exclusion respectively, also warrant prospective validation.

## Conclusions

In this retrospective study of emergency department patients with positive age-adjusted D-dimer results, the D-dimer/pCO₂ ratio demonstrated good diagnostic accuracy for stratifying PE risk, with an AUC of 0.811 and a negative predictive value of 96.4% at the optimal threshold of 44.91. Exploratory thresholds identified subgroups with no PE events observed below 18.1 and no central PE observed below 61.25 in this cohort. Although not suitable as a stand-alone rule-out test, this physiologically grounded ratio may help inform the imaging workflow in this defined context. Any clinical implementation would require prospective evaluation of the ratio as an add-on within validated PE diagnostic pathways (e.g., Wells/YEARS). Prospective, multicenter validation will be required to confirm generalizability and to define the role of the D-dimer/pCO₂ ratio in routine clinical pathways.

## Supplementary Information

Below is the link to the electronic supplementary material.

Supplementary Material 1

Supplementary Material 2

Supplementary Material 3

Supplementary Material 4

## Data Availability

The complete dataset and data dictionary are available from the corresponding author upon reasonable request.
