# Supplementary material for: Pre-CT risk stratification using the D-dimer/pCO₂ ratio in D-dimer–positive emergency department patients: diagnostic accuracy study
Source: BMC Emerg Med. 2025 Nov 17;25:237. doi: 10.1186/s12873-025-01395-6 (PMC12625727; doi:10.1186/s12873-025-01395-6)
Supplement: Supplementary file 1 — Supplementary Material 1 [file 12873_2025_1395_MOESM1_ESM.pdf]

Scale: D-DİMER/PCO2

Cutpoint	Sensitivity (%)	Specificity (%)	PPV (%)	NPV (%)	Youden's index	AUC	Metric Score
10.8199356913183	100%	0%	12.89%	NaN%	0.00000	0.811	1.000
11.2199036918138	100%	0.16%	12.91%	100%	0.00164	0.811	1.002
12.1098265895954	100%	0.33%	12.93%	100%	0.00329	0.811	1.003
12.2799097065463	100%	0.49%	12.95%	100%	0.00493	0.811	1.005
12.5263157894737	100%	0.66%	12.97%	100%	0.00658	0.811	1.007
12.636815920398	100%	0.82%	12.99%	100%	0.00822	0.811	1.008
12.8856624319419	100%	0.99%	13.01%	100%	0.00987	0.811	1.010
12.957264957265	100%	1.15%	13.02%	100%	0.01151	0.811	1.012
13.1027253668763	100%	1.32%	13.04%	100%	0.01316	0.811	1.013
13.1304347826087	100%	1.48%	13.06%	100%	0.01480	0.811	1.015
13.1639722863741	100%	1.64%	13.08%	100%	0.01645	0.811	1.016
13.1764705882353	100%	1.81%	13.1%	100%	0.01809	0.811	1.018
13.2112068965517	100%	1.97%	13.12%	100%	0.01974	0.811	1.020
13.4122287968442	100%	2.14%	13.14%	100%	0.02138	0.811	1.021
13.4252873563218	100%	2.3%	13.16%	100%	0.02303	0.811	1.023
13.444976076555	100%	2.47%	13.18%	100%	0.02467	0.811	1.025
13.5261194029851	100%	2.63%	13.2%	100%	0.02632	0.811	1.026
13.5322195704057	100%	2.8%	13.22%	100%	0.02796	0.811	1.028
13.6623376623377	100%	2.96%	13.24%	100%	0.02961	0.811	1.030
13.6725663716814	100%	3.12%	13.25%	100%	0.03125	0.811	1.031
13.7250554323725	100%	3.29%	13.27%	100%	0.03289	0.811	1.033
13.7391304347826	100%	3.45%	13.29%	100%	0.03454	0.811	1.035
13.8796680497925	100%	3.62%	13.31%	100%	0.03618	0.811	1.036
14.1176470588235	100%	3.78%	13.33%	100%	0.03783	0.811	1.038
14.1188524590164	100%	3.95%	13.35%	100%	0.03947	0.811	1.039
14.1794871794872	100%	4.11%	13.37%	100%	0.04112	0.811	1.041
14.2154566744731	100%	4.28%	13.39%	100%	0.04276	0.811	1.043
14.35960591133	100%	4.44%	13.41%	100%	0.04441	0.811	1.044
14.3914081145585	100%	4.61%	13.43%	100%	0.04605	0.811	1.046
14.572864321608	100%	4.77%	13.45%	100%	0.04770	0.811	1.048
14.8314606741573	100%	4.93%	13.47%	100%	0.04934	0.811	1.049
15.1304347826087	100%	5.1%	13.49%	100%	0.05099	0.811	1.051
15.4661016949153	100%	5.26%	13.51%	100%	0.05263	0.811	1.053
15.5741127348643	100%	5.43%	13.53%	100%	0.05428	0.811	1.054
15.6105610561056	100%	5.59%	13.55%	100%	0.05592	0.811	1.056
15.6315789473684	100%	5.76%	13.57%	100%	0.05757	0.811	1.058
15.692007797271	100%	5.92%	13.6%	100%	0.05921	0.811	1.059
15.8049886621315	100%	6.09%	13.62%	100%	0.06086	0.811	1.061
15.9061833688699	100%	6.25%	13.64%	100%	0.06250	0.811	1.063
15.9512195121951	100%	6.41%	13.66%	100%	0.06414	0.811	1.064
16.0084033613445	100%	6.58%	13.68%	100%	0.06579	0.811	1.066
16.0263157894737	100%	6.74%	13.7%	100%	0.06743	0.811	1.067
16.0483870967742	100%	6.91%	13.72%	100%	0.06908	0.811	1.069
16.0952380952381	100%	7.07%	13.74%	100%	0.07072	0.811	1.071
16.0986547085202	100%	7.24%	13.76%	100%	0.07237	0.811	1.072
16.1282051282051	100%	7.4%	13.78%	100%	0.07401	0.811	1.074
16.1662198391421	100%	7.57%	13.8%	100%	0.07566	0.811	1.076
16.2781954887218	100%	7.73%	13.82%	100%	0.07730	0.811	1.077
16.3466042154567	100%	7.89%	13.85%	100%	0.07895	0.811	1.079
16.4364640883978	100%	8.06%	13.87%	100%	0.08059	0.811	1.081

Scale: D-DİMER/PCO2

16.474358974359	100%	8.22%	13.89%	100%	0.08224	0.811	1.082
16.7331670822943	100%	8.39%	13.91%	100%	0.08388	0.811	1.084
16.7337807606264	100%	8.55%	13.93%	100%	0.08553	0.811	1.086
16.8181818181818	100%	8.72%	13.95%	100%	0.08717	0.811	1.087
16.8241469816273	100%	8.88%	13.98%	100%	0.08882	0.811	1.089
16.9248291571754	100%	9.05%	14%	100%	0.09046	0.811	1.090
16.9298245614035	100%	9.21%	14.02%	100%	0.09211	0.811	1.092
16.9402985074627	100%	9.38%	14.04%	100%	0.09375	0.811	1.094
16.9594594594595	100%	9.54%	14.06%	100%	0.09539	0.811	1.095
16.991643454039	100%	9.7%	14.08%	100%	0.09704	0.811	1.097
17.1867007672634	100%	9.87%	14.11%	100%	0.09868	0.811	1.099
17.2	100%	10.03%	14.13%	100%	0.10033	0.811	1.100
17.20823798627	100%	10.2%	14.15%	100%	0.10197	0.811	1.102
17.2549019607843	100%	10.36%	14.17%	100%	0.10362	0.811	1.104
17.2727272727273	100%	10.53%	14.2%	100%	0.10526	0.811	1.105
17.2986577181208	100%	10.69%	14.22%	100%	0.10691	0.811	1.107
17.34	100%	10.86%	14.24%	100%	0.10855	0.811	1.109
17.4285714285714	100%	11.02%	14.26%	100%	0.11020	0.811	1.110
17.4298056155508	100%	11.18%	14.29%	100%	0.11184	0.811	1.112
17.4352331606218	100%	11.35%	14.31%	100%	0.11349	0.811	1.113
17.4678111587983	100%	11.51%	14.33%	100%	0.11513	0.811	1.115
17.5	100%	11.68%	14.35%	100%	0.11678	0.811	1.117
17.5211267605634	100%	11.84%	14.38%	100%	0.11842	0.811	1.118
17.5510204081633	100%	12.01%	14.4%	100%	0.12007	0.811	1.120
17.6381909547739	100%	12.17%	14.42%	100%	0.12171	0.811	1.122
17.6437847866419	100%	12.34%	14.45%	100%	0.12336	0.811	1.123
17.6701570680628	100%	12.5%	14.47%	100%	0.12500	0.811	1.125
17.6781609195402	100%	12.66%	14.49%	100%	0.12664	0.811	1.127
17.7142857142857	100%	12.83%	14.52%	100%	0.12829	0.811	1.128
17.7530864197531	100%	12.99%	14.54%	100%	0.12993	0.811	1.130
17.787610619469	100%	13.16%	14.56%	100%	0.13158	0.811	1.132
17.8181818181818	100%	13.32%	14.59%	100%	0.13322	0.811	1.133
17.841726618705	100%	13.49%	14.61%	100%	0.13487	0.811	1.135
17.8961038961039	100%	13.65%	14.63%	100%	0.13651	0.811	1.137
17.921875	100%	13.82%	14.66%	100%	0.13816	0.811	1.138
17.9820627802691	100%	13.98%	14.68%	100%	0.13980	0.811	1.140
17.9879275653924	100%	14.14%	14.71%	100%	0.14145	0.811	1.141
17.9901960784314	100%	14.31%	14.73%	100%	0.14309	0.811	1.143
18.0449826989619	100%	14.47%	14.75%	100%	0.14474	0.811	1.145
18.0875202593193	100%	14.64%	14.78%	100%	0.14638	0.811	1.146
18.089552238806	100%	14.8%	14.8%	100%	0.14803	0.811	1.148
18.10888252149	100%	14.97%	14.83%	100%	0.14967	0.811	1.150
18.141592920354	98.89%	14.97%	14.69%	98.91%	0.13856	0.811	1.139
18.1497797356828	98.89%	15.13%	14.71%	98.92%	0.14020	0.811	1.140
18.4257206208426	98.89%	15.3%	14.74%	98.94%	0.14185	0.811	1.142
18.4517766497462	98.89%	15.46%	14.76%	98.95%	0.14349	0.811	1.143
18.452380952381	98.89%	15.62%	14.78%	98.96%	0.14514	0.811	1.145
18.5416666666667	98.89%	15.79%	14.81%	98.97%	0.14678	0.811	1.147
18.5539714867617	98.89%	15.95%	14.83%	98.98%	0.14843	0.811	1.148
18.5552407932011	98.89%	16.12%	14.86%	98.99%	0.15007	0.811	1.150
18.5828877005348	98.89%	16.28%	14.88%	99%	0.15172	0.811	1.152
18.7117903930131	98.89%	16.45%	14.91%	99.01%	0.15336	0.811	1.153
18.716049382716	98.89%	16.61%	14.93%	99.02%	0.15501	0.811	1.155

Scale: D-DİMER/PCO2

18.780487804878	98.89%	16.78%	14.96%	99.03%	0.15665	0.811	1.157
18.8183807439825	98.89%	16.94%	14.98%	99.04%	0.15830	0.811	1.158
18.8235294117647	98.89%	17.11%	15.01%	99.05%	0.15994	0.811	1.160
18.8612836438923	98.89%	17.27%	15.03%	99.06%	0.16159	0.811	1.162
18.8640973630832	98.89%	17.43%	15.06%	99.07%	0.16323	0.811	1.163
18.9592760180995	98.89%	17.6%	15.08%	99.07%	0.16488	0.811	1.165
18.9830508474576	98.89%	17.76%	15.11%	99.08%	0.16652	0.811	1.167
19.0184049079755	98.89%	17.93%	15.14%	99.09%	0.16817	0.811	1.168
19.0439770554493	98.89%	18.09%	15.16%	99.1%	0.16981	0.811	1.170
19.0724637681159	97.78%	18.09%	15.02%	98.21%	0.15870	0.811	1.159
19.0818858560794	97.78%	18.26%	15.04%	98.23%	0.16034	0.811	1.160
19.1085271317829	97.78%	18.42%	15.07%	98.25%	0.16199	0.811	1.162
19.1947565543071	97.78%	18.59%	15.09%	98.26%	0.16363	0.811	1.164
19.2050209205021	97.78%	18.75%	15.12%	98.28%	0.16528	0.811	1.165
19.3565683646113	97.78%	18.91%	15.15%	98.29%	0.16692	0.811	1.167
19.4019933554817	97.78%	19.08%	15.17%	98.31%	0.16857	0.811	1.169
19.4085027726433	97.78%	19.24%	15.2%	98.32%	0.17021	0.811	1.170
19.478021978022	97.78%	19.41%	15.22%	98.33%	0.17186	0.811	1.172
19.5032397408207	97.78%	19.57%	15.25%	98.35%	0.17350	0.811	1.174
19.504132231405	97.78%	19.74%	15.28%	98.36%	0.17515	0.811	1.175
19.5287958115183	97.78%	19.9%	15.3%	98.37%	0.17679	0.811	1.177
19.5377128953771	97.78%	20.07%	15.33%	98.39%	0.17844	0.811	1.178
19.6567505720824	97.78%	20.23%	15.36%	98.4%	0.18008	0.811	1.180
19.6626506024096	97.78%	20.39%	15.38%	98.41%	0.18173	0.811	1.182
19.7272727272727	97.78%	20.56%	15.41%	98.43%	0.18337	0.811	1.183
19.7820163487738	97.78%	20.72%	15.44%	98.44%	0.18501	0.811	1.185
19.8547215496368	97.78%	20.89%	15.47%	98.45%	0.18666	0.811	1.187
19.855421686747	97.78%	21.05%	15.49%	98.46%	0.18830	0.811	1.188
19.8606271777003	96.67%	21.05%	15.34%	97.71%	0.17719	0.811	1.177
19.9058380414313	96.67%	21.22%	15.37%	97.73%	0.17884	0.811	1.179
19.9698795180723	96.67%	21.38%	15.4%	97.74%	0.18048	0.811	1.180
20.1663893510815	96.67%	21.55%	15.43%	97.76%	0.18213	0.811	1.182
20.3287671232877	96.67%	21.71%	15.45%	97.78%	0.18377	0.811	1.184
20.3303303303303	96.67%	21.88%	15.48%	97.79%	0.18542	0.811	1.185
20.3703703703704	96.67%	22.04%	15.51%	97.81%	0.18706	0.811	1.187
20.4455445544554	96.67%	22.2%	15.54%	97.83%	0.18871	0.811	1.189
20.455764075067	96.67%	22.37%	15.56%	97.84%	0.19035	0.811	1.190
20.6306306306306	96.67%	22.53%	15.59%	97.86%	0.19200	0.811	1.192
20.7179487179487	96.67%	22.7%	15.62%	97.87%	0.19364	0.811	1.194
20.7266982622433	96.67%	22.86%	15.65%	97.89%	0.19529	0.811	1.195
20.7564575645756	96.67%	23.03%	15.68%	97.9%	0.19693	0.811	1.197
20.8478802992519	96.67%	23.19%	15.7%	97.92%	0.19857	0.811	1.199
20.8727272727273	96.67%	23.36%	15.73%	97.93%	0.20022	0.811	1.200
20.961145194274	96.67%	23.52%	15.76%	97.95%	0.20186	0.811	1.202
21.039755351682	96.67%	23.68%	15.79%	97.96%	0.20351	0.811	1.204
21.0765550239234	95.56%	23.68%	15.64%	97.3%	0.19240	0.811	1.192
21.2476722532588	95.56%	23.85%	15.66%	97.32%	0.19404	0.811	1.194
21.2605042016807	95.56%	24.01%	15.69%	97.33%	0.19569	0.811	1.196
21.2621359223301	95.56%	24.18%	15.72%	97.35%	0.19733	0.811	1.197
21.3380281690141	95.56%	24.34%	15.75%	97.37%	0.19898	0.811	1.199
21.3432835820896	95.56%	24.51%	15.78%	97.39%	0.20062	0.811	1.201
21.3532110091743	94.44%	24.51%	15.62%	96.75%	0.18951	0.811	1.190
21.3970588235294	94.44%	24.67%	15.65%	96.77%	0.19115	0.811	1.191

Scale: D-DİMER/PCO2

21.448275862069	93.33%	24.67%	15.5%	96.15%	0.18004	0.811	1.180
21.6071428571429	93.33%	24.84%	15.53%	96.18%	0.18169	0.811	1.182
21.6858237547893	93.33%	25%	15.56%	96.2%	0.18333	0.811	1.183
21.7391304347826	93.33%	25.16%	15.58%	96.23%	0.18498	0.811	1.185
21.7535545023697	93.33%	25.33%	15.61%	96.25%	0.18662	0.811	1.187
21.7811704834606	93.33%	25.49%	15.64%	96.27%	0.18827	0.811	1.188
21.8924731182796	93.33%	25.66%	15.67%	96.3%	0.18991	0.811	1.190
22.0815450643777	93.33%	25.82%	15.7%	96.32%	0.19156	0.811	1.192
22.0954907161804	93.33%	25.99%	15.73%	96.34%	0.19320	0.811	1.193
22.1292775665399	92.22%	25.99%	15.57%	95.76%	0.18209	0.811	1.182
22.2168674698795	92.22%	26.15%	15.6%	95.78%	0.18374	0.811	1.184
22.2356495468278	92.22%	26.32%	15.63%	95.81%	0.18538	0.811	1.185
22.2795698924731	92.22%	26.48%	15.66%	95.83%	0.18702	0.811	1.187
22.3062730627306	92.22%	26.64%	15.69%	95.86%	0.18867	0.811	1.189
22.375	92.22%	26.81%	15.72%	95.88%	0.19031	0.811	1.190
22.4220183486239	92.22%	26.97%	15.75%	95.91%	0.19196	0.811	1.192
22.4333333333333	92.22%	27.14%	15.78%	95.93%	0.19360	0.811	1.194
22.6588235294118	92.22%	27.3%	15.81%	95.95%	0.19525	0.811	1.195
22.8238341968912	92.22%	27.47%	15.84%	95.98%	0.19689	0.811	1.197
22.8482972136223	92.22%	27.63%	15.87%	96%	0.19854	0.811	1.199
22.8883495145631	92.22%	27.8%	15.9%	96.02%	0.20018	0.811	1.200
22.9032258064516	92.22%	27.96%	15.93%	96.05%	0.20183	0.811	1.202
22.903981264637	92.22%	28.12%	15.96%	96.07%	0.20347	0.811	1.203
22.9339853300734	92.22%	28.29%	15.99%	96.09%	0.20512	0.811	1.205
22.962962962963	92.22%	28.45%	16.02%	96.11%	0.20676	0.811	1.207
23.0618892508143	92.22%	28.62%	16.05%	96.13%	0.20841	0.811	1.208
23.0740740740741	92.22%	28.78%	16.09%	96.15%	0.21005	0.811	1.210
23.1552162849873	92.22%	28.95%	16.12%	96.17%	0.21170	0.811	1.212
23.2553191489362	92.22%	29.11%	16.15%	96.2%	0.21334	0.811	1.213
23.3407572383074	92.22%	29.28%	16.18%	96.22%	0.21499	0.811	1.215
23.3644859813084	92.22%	29.44%	16.21%	96.24%	0.21663	0.811	1.217
23.4924078091106	92.22%	29.61%	16.24%	96.26%	0.21827	0.811	1.218
23.5714285714286	92.22%	29.77%	16.27%	96.28%	0.21992	0.811	1.220
23.5787321063395	92.22%	29.93%	16.31%	96.3%	0.22156	0.811	1.222
23.7193763919822	92.22%	30.1%	16.34%	96.32%	0.22321	0.811	1.223
23.7264150943396	92.22%	30.26%	16.37%	96.34%	0.22485	0.811	1.225
23.7383177570093	92.22%	30.43%	16.4%	96.35%	0.22650	0.811	1.226
23.7558685446009	92.22%	30.59%	16.44%	96.37%	0.22814	0.811	1.228
23.8235294117647	92.22%	30.76%	16.47%	96.39%	0.22979	0.811	1.230
23.8477366255144	92.22%	30.92%	16.5%	96.41%	0.23143	0.811	1.231
23.8548752834467	92.22%	31.09%	16.53%	96.43%	0.23308	0.811	1.233
23.9007092198582	92.22%	31.25%	16.57%	96.45%	0.23472	0.811	1.235
24.0960451977401	92.22%	31.41%	16.6%	96.46%	0.23637	0.811	1.236
24.1114058355438	92.22%	31.58%	16.63%	96.48%	0.23801	0.811	1.238
24.2025862068966	91.11%	31.58%	16.47%	96%	0.22690	0.811	1.227
24.4	91.11%	31.74%	16.5%	96.02%	0.22855	0.811	1.229
24.4309927360775	91.11%	31.91%	16.53%	96.04%	0.23019	0.811	1.230
24.4487427466151	91.11%	32.07%	16.57%	96.06%	0.23183	0.811	1.232
24.4796380090498	91.11%	32.24%	16.6%	96.08%	0.23348	0.811	1.233
24.5244956772334	91.11%	32.4%	16.63%	96.1%	0.23512	0.811	1.235
24.6578947368421	91.11%	32.57%	16.67%	96.12%	0.23677	0.811	1.237
24.688995215311	91.11%	32.73%	16.7%	96.14%	0.23841	0.811	1.238
24.7545219638243	91.11%	32.89%	16.73%	96.15%	0.24006	0.811	1.240

24.7674418604651	91.11%	33.06%	16.77%	96.17%	0.24170	0.811	1.242
24.8214285714286	91.11%	33.22%	16.8%	96.19%	0.24335	0.811	1.243
24.8536585365854	91.11%	33.39%	16.84%	96.21%	0.24499	0.811	1.245
24.915611814346	91.11%	33.55%	16.87%	96.23%	0.24664	0.811	1.247
24.9175824175824	91.11%	33.72%	16.91%	96.24%	0.24828	0.811	1.248
24.9289099526066	91.11%	33.88%	16.94%	96.26%	0.24993	0.811	1.250
24.9465240641711	91.11%	34.05%	16.98%	96.28%	0.25157	0.811	1.252
24.965034965035	91.11%	34.21%	17.01%	96.3%	0.25322	0.811	1.253
24.9761904761905	91.11%	34.38%	17.05%	96.31%	0.25486	0.811	1.255
24.9882352941176	91.11%	34.54%	17.08%	96.33%	0.25651	0.811	1.257
25	90%	34.54%	16.91%	95.89%	0.24539	0.811	1.245
25.0147928994083	90%	34.7%	16.95%	95.91%	0.24704	0.811	1.247
25.1653944020356	90%	34.87%	16.98%	95.93%	0.24868	0.811	1.249
25.2300242130751	90%	35.03%	17.02%	95.95%	0.25033	0.811	1.250
25.4166666666667	90%	35.2%	17.05%	95.96%	0.25197	0.811	1.252
25.4460093896714	90%	35.36%	17.09%	95.98%	0.25362	0.811	1.254
25.4741379310345	90%	35.53%	17.12%	96%	0.25526	0.811	1.255
25.531914893617	90%	35.69%	17.16%	96.02%	0.25691	0.811	1.257
25.5351681957187	90%	35.86%	17.2%	96.04%	0.25855	0.811	1.259
25.5625	90%	36.02%	17.23%	96.05%	0.26020	0.811	1.260
25.5690072639225	90%	36.18%	17.27%	96.07%	0.26184	0.811	1.262
25.5882352941176	90%	36.35%	17.31%	96.09%	0.26349	0.811	1.263
25.6300268096515	90%	36.51%	17.34%	96.1%	0.26513	0.811	1.265
25.7824933687003	90%	36.68%	17.38%	96.12%	0.26678	0.811	1.267
25.800464037123	90%	36.84%	17.42%	96.14%	0.26842	0.811	1.268
25.8208955223881	90%	37.01%	17.46%	96.15%	0.27007	0.811	1.270
25.8415841584158	90%	37.17%	17.49%	96.17%	0.27171	0.811	1.272
25.8595641646489	90%	37.34%	17.53%	96.19%	0.27336	0.811	1.273
25.8785249457701	90%	37.5%	17.57%	96.2%	0.27500	0.811	1.275
25.9751037344398	90%	37.66%	17.61%	96.22%	0.27664	0.811	1.277
26.0043668122271	90%	37.83%	17.65%	96.23%	0.27829	0.811	1.278
26.0722891566265	90%	37.99%	17.69%	96.25%	0.27993	0.811	1.280
26.0801781737194	90%	38.16%	17.72%	96.27%	0.28158	0.811	1.282
26.1096605744125	90%	38.32%	17.76%	96.28%	0.28322	0.811	1.283
26.1467889908257	90%	38.49%	17.8%	96.3%	0.28487	0.811	1.285
26.1715481171548	90%	38.65%	17.84%	96.31%	0.28651	0.811	1.287
26.2368421052632	90%	38.82%	17.88%	96.33%	0.28816	0.811	1.288
26.2385321100917	90%	38.98%	17.92%	96.34%	0.28980	0.811	1.290
26.3013698630137	90%	39.14%	17.96%	96.36%	0.29145	0.811	1.291
26.5029469548134	90%	39.31%	18%	96.37%	0.29309	0.811	1.293
26.5131578947368	90%	39.47%	18.04%	96.39%	0.29474	0.811	1.295
26.5333333333333	90%	39.64%	18.08%	96.4%	0.29638	0.811	1.296
26.5860215053763	90%	39.8%	18.12%	96.41%	0.29803	0.811	1.298
26.6164154103853	90%	39.97%	18.16%	96.43%	0.29967	0.811	1.300
26.6835443037975	88.89%	39.97%	17.98%	96.05%	0.28856	0.811	1.289
26.7037861915368	88.89%	40.13%	18.02%	96.06%	0.29020	0.811	1.290
26.9463087248322	88.89%	40.3%	18.06%	96.08%	0.29185	0.811	1.292
26.9889502762431	88.89%	40.46%	18.1%	96.09%	0.29349	0.811	1.293
27.0676691729323	88.89%	40.62%	18.14%	96.11%	0.29514	0.811	1.295
27.1194379391101	88.89%	40.79%	18.18%	96.12%	0.29678	0.811	1.297
27.1230158730159	88.89%	40.95%	18.22%	96.14%	0.29843	0.811	1.298
27.1276595744681	88.89%	41.12%	18.26%	96.15%	0.30007	0.811	1.300
27.1304347826087	88.89%	41.28%	18.31%	96.17%	0.30172	0.811	1.302

27.1363636363636	88.89%	41.45%	18.35%	96.18%	0.30336	0.811	1.303
27.2768878718535	88.89%	41.61%	18.39%	96.2%	0.30501	0.811	1.305
27.3414634146341	88.89%	41.78%	18.43%	96.21%	0.30665	0.811	1.307
27.3655913978495	88.89%	41.94%	18.48%	96.23%	0.30830	0.811	1.308
27.3700305810398	88.89%	42.11%	18.52%	96.24%	0.30994	0.811	1.310
27.4025974025974	88.89%	42.27%	18.56%	96.25%	0.31159	0.811	1.312
27.4260355029586	88.89%	42.43%	18.6%	96.27%	0.31323	0.811	1.313
27.43	88.89%	42.6%	18.65%	96.28%	0.31488	0.811	1.315
27.5268817204301	88.89%	42.76%	18.69%	96.3%	0.31652	0.811	1.317
27.7777777777778	88.89%	42.93%	18.74%	96.31%	0.31817	0.811	1.318
27.8228782287823	88.89%	43.09%	18.78%	96.32%	0.31981	0.811	1.320
27.8772378516624	88.89%	43.26%	18.82%	96.34%	0.32145	0.811	1.321
27.9057591623037	88.89%	43.42%	18.87%	96.35%	0.32310	0.811	1.323
27.9190751445087	88.89%	43.59%	18.91%	96.36%	0.32474	0.811	1.325
28.1185567010309	88.89%	43.75%	18.96%	96.38%	0.32639	0.811	1.326
28.125	88.89%	43.91%	19%	96.39%	0.32803	0.811	1.328
28.1542056074766	88.89%	44.08%	19.05%	96.4%	0.32968	0.811	1.330
28.1571428571429	88.89%	44.24%	19.09%	96.42%	0.33132	0.811	1.331
28.3238636363636	88.89%	44.41%	19.14%	96.43%	0.33297	0.811	1.333
28.3253588516746	88.89%	44.57%	19.18%	96.44%	0.33461	0.811	1.335
28.4894837476099	88.89%	44.74%	19.23%	96.45%	0.33626	0.811	1.336
28.5714285714286	88.89%	44.9%	19.28%	96.47%	0.33790	0.811	1.338
28.6545454545455	88.89%	45.07%	19.32%	96.48%	0.33955	0.811	1.340
28.711943793911	88.89%	45.23%	19.37%	96.49%	0.34119	0.811	1.341
28.8582677165354	88.89%	45.39%	19.42%	96.5%	0.34284	0.811	1.343
28.8642659279778	88.89%	45.56%	19.46%	96.52%	0.34448	0.811	1.344
28.9819004524887	88.89%	45.72%	19.51%	96.53%	0.34613	0.811	1.346
28.9893617021277	88.89%	45.89%	19.56%	96.54%	0.34777	0.811	1.348
29.0461997019374	88.89%	46.05%	19.61%	96.55%	0.34942	0.811	1.349
29.0748898678414	88.89%	46.22%	19.66%	96.56%	0.35106	0.811	1.351
29.1136363636364	88.89%	46.38%	19.7%	96.58%	0.35270	0.811	1.353
29.1150442477876	88.89%	46.55%	19.75%	96.59%	0.35435	0.811	1.354
29.1412742382271	88.89%	46.71%	19.8%	96.6%	0.35599	0.811	1.356
29.3017456359102	88.89%	46.88%	19.85%	96.61%	0.35764	0.811	1.358
29.4858156028369	88.89%	47.04%	19.9%	96.62%	0.35928	0.811	1.359
29.5529411764706	88.89%	47.2%	19.95%	96.63%	0.36093	0.811	1.361
29.725	88.89%	47.37%	20%	96.64%	0.36257	0.811	1.363
29.7385620915033	88.89%	47.53%	20.05%	96.66%	0.36422	0.811	1.364
29.7527472527473	88.89%	47.7%	20.1%	96.67%	0.36586	0.811	1.366
29.7984886649874	88.89%	47.86%	20.15%	96.68%	0.36751	0.811	1.368
29.8123324396783	87.78%	47.86%	19.95%	96.36%	0.35640	0.811	1.356
29.8257080610022	87.78%	48.03%	20%	96.37%	0.35804	0.811	1.358
29.9418604651163	87.78%	48.19%	20.05%	96.38%	0.35969	0.811	1.360
30.0242718446602	87.78%	48.36%	20.1%	96.39%	0.36133	0.811	1.361
30.1814516129032	87.78%	48.52%	20.15%	96.41%	0.36298	0.811	1.363
30.183299389002	87.78%	48.68%	20.2%	96.42%	0.36462	0.811	1.365
30.3009259259259	87.78%	48.85%	20.26%	96.43%	0.36626	0.811	1.366
30.3174603174603	87.78%	49.01%	20.31%	96.44%	0.36791	0.811	1.368
30.6	87.78%	49.18%	20.36%	96.45%	0.36955	0.811	1.370
30.613810741688	87.78%	49.34%	20.41%	96.46%	0.37120	0.811	1.371
30.6474820143885	87.78%	49.51%	20.47%	96.47%	0.37284	0.811	1.373
30.7075471698113	87.78%	49.67%	20.52%	96.49%	0.37449	0.811	1.374
30.7377049180328	87.78%	49.84%	20.57%	96.5%	0.37613	0.811	1.376

Scale: D-DİMER/PCO2

30.7600950118765	87.78%	50%	20.63%	96.51%	0.37778	0.811	1.378
30.8208955223881	87.78%	50.16%	20.68%	96.52%	0.37942	0.811	1.379
30.8396946564886	87.78%	50.33%	20.73%	96.53%	0.38107	0.811	1.381
30.873786407767	87.78%	50.49%	20.79%	96.54%	0.38271	0.811	1.383
31.1764705882353	87.78%	50.66%	20.84%	96.55%	0.38436	0.811	1.384
31.2190082644628	87.78%	50.82%	20.9%	96.56%	0.38600	0.811	1.386
31.2389380530973	87.78%	50.99%	20.95%	96.57%	0.38765	0.811	1.388
31.3480392156863	87.78%	51.15%	21.01%	96.58%	0.38929	0.811	1.389
31.4565826330532	87.78%	51.32%	21.07%	96.59%	0.39094	0.811	1.391
31.5667311411992	87.78%	51.48%	21.12%	96.6%	0.39258	0.811	1.393
31.5722120658135	87.78%	51.64%	21.18%	96.62%	0.39423	0.811	1.394
31.5909090909091	87.78%	51.81%	21.24%	96.63%	0.39587	0.811	1.396
31.6754850088183	87.78%	51.97%	21.29%	96.64%	0.39751	0.811	1.398
31.6847826086957	87.78%	52.14%	21.35%	96.65%	0.39916	0.811	1.399
31.9723183391003	87.78%	52.3%	21.41%	96.66%	0.40080	0.811	1.401
31.9954648526077	87.78%	52.47%	21.47%	96.67%	0.40245	0.811	1.402
32.1441124780316	87.78%	52.63%	21.53%	96.68%	0.40409	0.811	1.404
32.2588235294118	87.78%	52.8%	21.58%	96.69%	0.40574	0.811	1.406
32.3770491803279	87.78%	52.96%	21.64%	96.7%	0.40738	0.811	1.407
32.4545454545455	87.78%	53.12%	21.7%	96.71%	0.40903	0.811	1.409
32.4931506849315	87.78%	53.29%	21.76%	96.72%	0.41067	0.811	1.411
32.5770308123249	87.78%	53.45%	21.82%	96.73%	0.41232	0.811	1.412
32.5862068965517	87.78%	53.62%	21.88%	96.74%	0.41396	0.811	1.414
32.7083333333333	87.78%	53.78%	21.94%	96.75%	0.41561	0.811	1.416
32.7710843373494	87.78%	53.95%	22.01%	96.76%	0.41725	0.811	1.417
32.7835051546392	87.78%	54.11%	22.07%	96.76%	0.41890	0.811	1.419
32.8518518518519	87.78%	54.28%	22.13%	96.77%	0.42054	0.811	1.421
33.1111111111111	87.78%	54.44%	22.19%	96.78%	0.42219	0.811	1.422
33.2960893854749	87.78%	54.61%	22.25%	96.79%	0.42383	0.811	1.424
33.5149863760218	87.78%	54.77%	22.32%	96.8%	0.42548	0.811	1.425
33.5421166306695	87.78%	54.93%	22.38%	96.81%	0.42712	0.811	1.427
33.5526315789474	87.78%	55.1%	22.44%	96.82%	0.42876	0.811	1.429
33.5693215339233	87.78%	55.26%	22.51%	96.83%	0.43041	0.811	1.430
33.5772357723577	87.78%	55.43%	22.57%	96.84%	0.43205	0.811	1.432
33.7536656891496	87.78%	55.59%	22.64%	96.85%	0.43370	0.811	1.434
33.7783375314861	87.78%	55.76%	22.7%	96.86%	0.43534	0.811	1.435
33.9800995024876	86.67%	55.76%	22.48%	96.58%	0.42423	0.811	1.424
34.0992167101828	86.67%	55.92%	22.54%	96.59%	0.42588	0.811	1.426
34.1312056737589	86.67%	56.09%	22.61%	96.6%	0.42752	0.811	1.428
34.188790560472	86.67%	56.25%	22.67%	96.61%	0.42917	0.811	1.429
34.2105263157895	86.67%	56.41%	22.74%	96.62%	0.43081	0.811	1.431
34.2253521126761	86.67%	56.58%	22.81%	96.63%	0.43246	0.811	1.432
34.2595978062157	86.67%	56.74%	22.87%	96.64%	0.43410	0.811	1.434
34.2857142857143	86.67%	56.91%	22.94%	96.65%	0.43575	0.811	1.436
34.4242424242424	86.67%	57.07%	23.01%	96.66%	0.43739	0.811	1.437
34.5185185185185	86.67%	57.24%	23.08%	96.67%	0.43904	0.811	1.439
34.7529411764706	86.67%	57.4%	23.15%	96.68%	0.44068	0.811	1.441
34.8	86.67%	57.57%	23.21%	96.69%	0.44232	0.811	1.442
34.8816568047337	86.67%	57.73%	23.28%	96.69%	0.44397	0.811	1.444
34.9049429657795	86.67%	57.89%	23.35%	96.7%	0.44561	0.811	1.446
34.9063670411985	86.67%	58.06%	23.42%	96.71%	0.44726	0.811	1.447
35.0928381962865	86.67%	58.22%	23.49%	96.72%	0.44890	0.811	1.449
35.2823920265781	86.67%	58.39%	23.56%	96.73%	0.45055	0.811	1.451

Scale: D-DİMER/PCO2

35.6119402985075	86.67%	58.55%	23.64%	96.74%	0.45219	0.811	1.452
35.8287795992714	85.56%	58.55%	23.4%	96.48%	0.44108	0.811	1.441
35.8659217877095	85.56%	58.72%	23.48%	96.49%	0.44273	0.811	1.443
35.9910913140312	85.56%	58.88%	23.55%	96.5%	0.44437	0.811	1.444
36.0722891566265	85.56%	59.05%	23.62%	96.51%	0.44602	0.811	1.446
36.25	85.56%	59.21%	23.69%	96.51%	0.44766	0.811	1.448
36.2531017369727	85.56%	59.38%	23.77%	96.52%	0.44931	0.811	1.449
36.2569832402235	85.56%	59.54%	23.84%	96.53%	0.45095	0.811	1.451
36.3488372093023	85.56%	59.7%	23.91%	96.54%	0.45260	0.811	1.453
36.3982102908277	85.56%	59.87%	23.99%	96.55%	0.45424	0.811	1.454
36.4110429447853	85.56%	60.03%	24.06%	96.56%	0.45588	0.811	1.456
36.4353312302839	85.56%	60.2%	24.14%	96.57%	0.45753	0.811	1.458
36.4490861618799	85.56%	60.36%	24.21%	96.58%	0.45917	0.811	1.459
36.5507246376812	85.56%	60.53%	24.29%	96.59%	0.46082	0.811	1.461
36.7025862068966	85.56%	60.69%	24.37%	96.6%	0.46246	0.811	1.462
36.8238993710692	85.56%	60.86%	24.44%	96.61%	0.46411	0.811	1.464
36.916890080429	85.56%	61.02%	24.52%	96.61%	0.46575	0.811	1.466
37	85.56%	61.18%	24.6%	96.62%	0.46740	0.811	1.467
37.2378516624041	85.56%	61.35%	24.68%	96.63%	0.46904	0.811	1.469
37.3233404710921	85.56%	61.51%	24.76%	96.64%	0.47069	0.811	1.471
37.4055415617128	85.56%	61.68%	24.84%	96.65%	0.47233	0.811	1.472
37.488038277512	85.56%	61.84%	24.92%	96.66%	0.47398	0.811	1.474
37.5466666666667	85.56%	62.01%	25%	96.67%	0.47562	0.811	1.476
37.6599063962559	85.56%	62.17%	25.08%	96.68%	0.47727	0.811	1.477
37.7941176470588	85.56%	62.34%	25.16%	96.68%	0.47891	0.811	1.479
38	85.56%	62.5%	25.25%	96.69%	0.48056	0.811	1.481
38.1774580335731	85.56%	62.66%	25.33%	96.7%	0.48220	0.811	1.482
38.2920792079208	85.56%	62.83%	25.41%	96.71%	0.48385	0.811	1.484
38.34	85.56%	62.99%	25.5%	96.72%	0.48549	0.811	1.485
38.3555555555556	85.56%	63.16%	25.58%	96.73%	0.48713	0.811	1.487
38.4275184275184	85.56%	63.32%	25.67%	96.73%	0.48878	0.811	1.489
38.4600760456274	85.56%	63.49%	25.75%	96.74%	0.49042	0.811	1.490
38.54	84.44%	63.49%	25.5%	96.5%	0.47931	0.811	1.479
38.6453201970443	84.44%	63.65%	25.59%	96.51%	0.48096	0.811	1.481
38.6567164179104	84.44%	63.82%	25.68%	96.52%	0.48260	0.811	1.483
38.75	84.44%	63.98%	25.76%	96.53%	0.48425	0.811	1.484
38.7719298245614	84.44%	64.14%	25.85%	96.53%	0.48589	0.811	1.486
39.0361445783133	84.44%	64.31%	25.94%	96.54%	0.48754	0.811	1.488
39.0728476821192	84.44%	64.47%	26.03%	96.55%	0.48918	0.811	1.489
39.08	84.44%	64.64%	26.12%	96.56%	0.49083	0.811	1.491
39.1002570694087	84.44%	64.8%	26.21%	96.57%	0.49247	0.811	1.492
39.125	84.44%	64.97%	26.3%	96.58%	0.49412	0.811	1.494
39.5043731778426	84.44%	65.13%	26.39%	96.59%	0.49576	0.811	1.496
40.3783783783784	84.44%	65.3%	26.48%	96.59%	0.49740	0.811	1.497
40.7142857142857	84.44%	65.46%	26.57%	96.6%	0.49905	0.811	1.499
40.8219178082192	84.44%	65.62%	26.67%	96.61%	0.50069	0.811	1.501
41.020942408377	84.44%	65.79%	26.76%	96.62%	0.50234	0.811	1.502
41.0334346504559	84.44%	65.95%	26.86%	96.63%	0.50398	0.811	1.504
41.5222482435597	84.44%	66.12%	26.95%	96.63%	0.50563	0.811	1.506
41.7204301075269	84.44%	66.28%	27.05%	96.64%	0.50727	0.811	1.507
41.8588235294118	84.44%	66.45%	27.14%	96.65%	0.50892	0.811	1.509
41.9315403422983	84.44%	66.61%	27.24%	96.66%	0.51056	0.811	1.511
41.9402985074627	84.44%	66.78%	27.34%	96.67%	0.51221	0.811	1.512

41.9714964370546	84.44%	66.94%	27.44%	96.67%	0.51385	0.811	1.514
42.0689655172414	84.44%	67.11%	27.54%	96.68%	0.51550	0.811	1.515
42.1375921375921	84.44%	67.27%	27.64%	96.69%	0.51714	0.811	1.517
42.1739130434783	84.44%	67.43%	27.74%	96.7%	0.51879	0.811	1.519
42.2796352583587	84.44%	67.6%	27.84%	96.71%	0.52043	0.811	1.520
42.3512747875354	84.44%	67.76%	27.94%	96.71%	0.52208	0.811	1.522
42.434554973822	84.44%	67.93%	28.04%	96.72%	0.52372	0.811	1.524
42.5449101796407	83.33%	67.93%	27.78%	96.5%	0.51261	0.811	1.513
42.5566343042071	83.33%	68.09%	27.88%	96.5%	0.51425	0.811	1.514
42.5816023738872	83.33%	68.26%	27.99%	96.51%	0.51590	0.811	1.516
42.6004728132388	83.33%	68.42%	28.09%	96.52%	0.51754	0.811	1.518
42.6395939086294	83.33%	68.59%	28.2%	96.53%	0.51919	0.811	1.519
42.9963235294118	82.22%	68.59%	27.92%	96.3%	0.50808	0.811	1.508
43.3060109289617	82.22%	68.75%	28.03%	96.31%	0.50972	0.811	1.510
43.5103244837758	82.22%	68.91%	28.14%	96.32%	0.51137	0.811	1.511
43.5227272727273	82.22%	69.08%	28.24%	96.33%	0.51301	0.811	1.513
43.7784090909091	82.22%	69.24%	28.35%	96.34%	0.51466	0.811	1.515
43.8888888888889	82.22%	69.41%	28.46%	96.35%	0.51630	0.811	1.516
44.0810810810811	82.22%	69.57%	28.57%	96.36%	0.51795	0.811	1.518
44.1685649202733	82.22%	69.74%	28.68%	96.36%	0.51959	0.811	1.520
44.2333333333333	82.22%	69.9%	28.79%	96.37%	0.52124	0.811	1.521
44.3204868154158	82.22%	70.07%	28.91%	96.38%	0.52288	0.811	1.523
44.3582887700535	82.22%	70.23%	29.02%	96.39%	0.52452	0.811	1.525
44.3922651933702	82.22%	70.39%	29.13%	96.4%	0.52617	0.811	1.526
44.4496487119438	82.22%	70.56%	29.25%	96.4%	0.52781	0.811	1.528
44.5301204819277	82.22%	70.72%	29.37%	96.41%	0.52946	0.811	1.529
44.7948717948718	82.22%	70.89%	29.48%	96.42%	0.53110	0.811	1.531
44.9101796407186	82.22%	71.05%	29.6%	96.43%	0.53275	0.811	1.533
44.9786324786325	81.11%	71.05%	29.32%	96.21%	0.52164	0.811	1.522
45.0749063670412	81.11%	71.22%	29.44%	96.22%	0.52328	0.811	1.523
45.2091254752852	80%	71.22%	29.15%	96.01%	0.51217	0.811	1.512
45.4245283018868	80%	71.38%	29.27%	96.02%	0.51382	0.811	1.514
45.5081967213115	80%	71.55%	29.39%	96.03%	0.51546	0.811	1.515
45.5298013245033	80%	71.71%	29.51%	96.04%	0.51711	0.811	1.517
45.5361596009975	78.89%	71.71%	29.22%	95.82%	0.50599	0.811	1.506
45.6666666666667	78.89%	71.88%	29.34%	95.83%	0.50764	0.811	1.508
45.7110609480813	78.89%	72.04%	29.46%	95.84%	0.50928	0.811	1.509
45.9101654846336	77.78%	72.04%	29.17%	95.63%	0.49817	0.811	1.498
46.0127931769723	77.78%	72.2%	29.29%	95.64%	0.49982	0.811	1.500
46.0436893203883	77.78%	72.37%	29.41%	95.65%	0.50146	0.811	1.501
46.3356164383562	77.78%	72.53%	29.54%	95.66%	0.50311	0.811	1.503
46.4383561643836	77.78%	72.7%	29.66%	95.67%	0.50475	0.811	1.505
47.2853828306264	76.67%	72.7%	29.36%	95.46%	0.49364	0.811	1.494
47.3618090452261	76.67%	72.86%	29.49%	95.47%	0.49529	0.811	1.495
47.3939393939394	76.67%	73.03%	29.61%	95.48%	0.49693	0.811	1.497
48.2198952879581	76.67%	73.19%	29.74%	95.49%	0.49857	0.811	1.499
48.3636363636364	76.67%	73.36%	29.87%	95.5%	0.50022	0.811	1.500
48.5398230088496	76.67%	73.52%	30%	95.51%	0.50186	0.811	1.502
48.6073059360731	76.67%	73.68%	30.13%	95.52%	0.50351	0.811	1.504
48.6526315789474	76.67%	73.85%	30.26%	95.53%	0.50515	0.811	1.505
48.6915887850467	76.67%	74.01%	30.4%	95.54%	0.50680	0.811	1.507
48.7031700288184	76.67%	74.18%	30.53%	95.55%	0.50844	0.811	1.508
49.5463510848126	76.67%	74.34%	30.67%	95.56%	0.51009	0.811	1.510

Scale: D-DİMER/PCO2

49.7959183673469	76.67%	74.51%	30.8%	95.57%	0.51173	0.811	1.512
49.9563318777293	76.67%	74.67%	30.94%	95.58%	0.51338	0.811	1.513
49.9719887955182	76.67%	74.84%	31.08%	95.59%	0.51502	0.811	1.515
50.0183486238532	76.67%	75%	31.22%	95.6%	0.51667	0.811	1.517
50.1285347043702	75.56%	75%	30.91%	95.4%	0.50556	0.811	1.506
50.1666666666667	74.44%	75%	30.59%	95.2%	0.49444	0.811	1.494
50.3405572755418	74.44%	75.16%	30.73%	95.21%	0.49609	0.811	1.496
50.6732673267327	73.33%	75.16%	30.41%	95.01%	0.48498	0.811	1.485
50.6808510638298	73.33%	75.33%	30.56%	95.02%	0.48662	0.811	1.487
51.3447432762836	73.33%	75.49%	30.7%	95.03%	0.48827	0.811	1.488
51.4512471655329	73.33%	75.66%	30.84%	95.04%	0.48991	0.811	1.490
51.6040100250627	73.33%	75.82%	30.99%	95.05%	0.49156	0.811	1.492
51.6258351893096	73.33%	75.99%	31.13%	95.06%	0.49320	0.811	1.493
51.7538461538462	73.33%	76.15%	31.28%	95.07%	0.49485	0.811	1.495
51.9111111111111	73.33%	76.32%	31.43%	95.08%	0.49649	0.811	1.496
52.0776255707763	72.22%	76.32%	31.1%	94.89%	0.48538	0.811	1.485
52.1487603305785	72.22%	76.48%	31.25%	94.9%	0.48702	0.811	1.487
52.6719576719577	72.22%	76.64%	31.4%	94.91%	0.48867	0.811	1.489
52.807881773399	72.22%	76.81%	31.55%	94.92%	0.49031	0.811	1.490
53.1536388140162	72.22%	76.97%	31.71%	94.93%	0.49196	0.811	1.492
53.8533834586466	72.22%	77.14%	31.86%	94.94%	0.49360	0.811	1.494
54.8192771084337	72.22%	77.3%	32.02%	94.95%	0.49525	0.811	1.495
55.7366771159875	72.22%	77.47%	32.18%	94.96%	0.49689	0.811	1.497
56.4724919093851	72.22%	77.63%	32.34%	94.97%	0.49854	0.811	1.499
56.8571428571429	72.22%	77.8%	32.5%	94.98%	0.50018	0.811	1.500
57.3780487804878	72.22%	77.96%	32.66%	94.99%	0.50183	0.811	1.502
57.4821852731591	72.22%	78.12%	32.83%	95%	0.50347	0.811	1.503
57.7536231884058	72.22%	78.29%	32.99%	95.01%	0.50512	0.811	1.505
57.9445727482679	72.22%	78.45%	33.16%	95.02%	0.50676	0.811	1.507
57.97783933518	72.22%	78.62%	33.33%	95.03%	0.50841	0.811	1.508
58.1303116147309	72.22%	78.78%	33.51%	95.04%	0.51005	0.811	1.510
58.1440443213296	72.22%	78.95%	33.68%	95.05%	0.51170	0.811	1.512
58.5675675675676	72.22%	79.11%	33.85%	95.06%	0.51334	0.811	1.513
58.938679245283	72.22%	79.28%	34.03%	95.07%	0.51499	0.811	1.515
59.0818858560794	72.22%	79.44%	34.21%	95.08%	0.51663	0.811	1.517
60.027397260274	72.22%	79.61%	34.39%	95.09%	0.51827	0.811	1.518
60.1069518716578	72.22%	79.77%	34.57%	95.1%	0.51992	0.811	1.520
60.2179176755448	72.22%	79.93%	34.76%	95.11%	0.52156	0.811	1.522
60.2640264026403	72.22%	80.1%	34.95%	95.12%	0.52321	0.811	1.523
60.4872881355932	72.22%	80.26%	35.14%	95.13%	0.52485	0.811	1.525
60.7042253521127	71.11%	80.26%	34.78%	94.94%	0.51374	0.811	1.514
61.2518628912072	71.11%	80.43%	34.97%	94.95%	0.51539	0.811	1.515
61.275	70%	80.43%	34.62%	94.77%	0.50428	0.811	1.504
61.4590747330961	70%	80.59%	34.81%	94.78%	0.50592	0.811	1.506
61.7054263565891	70%	80.76%	35%	94.79%	0.50757	0.811	1.508
61.8895348837209	68.89%	80.76%	34.64%	94.61%	0.49645	0.811	1.496
62.5925925925926	68.89%	80.92%	34.83%	94.62%	0.49810	0.811	1.498
62.7327327327327	68.89%	81.09%	35.03%	94.63%	0.49974	0.811	1.500
63.9400921658986	67.78%	81.09%	34.66%	94.44%	0.48863	0.811	1.489
64.1304347826087	67.78%	81.25%	34.86%	94.46%	0.49028	0.811	1.490
64.4946808510638	67.78%	81.41%	35.06%	94.47%	0.49192	0.811	1.492
65.1980198019802	66.67%	81.41%	34.68%	94.29%	0.48081	0.811	1.481
65.2579034941764	66.67%	81.58%	34.88%	94.3%	0.48246	0.811	1.482

65.2957746478873	66.67%	81.74%	35.09%	94.31%	0.48410	0.811	1.484
65.5365853658537	66.67%	81.91%	35.29%	94.32%	0.48575	0.811	1.486
65.6010230179028	65.56%	81.91%	34.91%	94.14%	0.47463	0.811	1.475
67.202216066482	65.56%	82.07%	35.12%	94.15%	0.47628	0.811	1.476
67.3913043478261	65.56%	82.24%	35.33%	94.16%	0.47792	0.811	1.478
67.4172185430464	65.56%	82.4%	35.54%	94.17%	0.47957	0.811	1.480
67.9669030732861	65.56%	82.57%	35.76%	94.18%	0.48121	0.811	1.481
68.0769230769231	64.44%	82.57%	35.37%	94.01%	0.47010	0.811	1.470
69.3714285714286	64.44%	82.73%	35.58%	94.02%	0.47175	0.811	1.472
70.3076923076923	64.44%	82.89%	35.8%	94.03%	0.47339	0.811	1.473
70.4129793510324	64.44%	83.06%	36.02%	94.04%	0.47504	0.811	1.475
70.7744107744108	64.44%	83.22%	36.25%	94.05%	0.47668	0.811	1.477
72.4701195219124	64.44%	83.39%	36.48%	94.06%	0.47833	0.811	1.478
73.9572192513369	64.44%	83.55%	36.71%	94.07%	0.47997	0.811	1.480
74.4444444444444	63.33%	83.55%	36.31%	93.9%	0.46886	0.811	1.469
74.9015317286652	63.33%	83.72%	36.54%	93.91%	0.47050	0.811	1.471
75.3658536585366	63.33%	83.88%	36.77%	93.92%	0.47215	0.811	1.472
75.6120527306968	62.22%	83.88%	36.36%	93.75%	0.46104	0.811	1.461
75.8761061946903	62.22%	84.05%	36.6%	93.76%	0.46268	0.811	1.463
77.3493975903614	62.22%	84.21%	36.84%	93.77%	0.46433	0.811	1.464
77.7333333333333	62.22%	84.38%	37.09%	93.78%	0.46597	0.811	1.466
78.2820512820513	62.22%	84.54%	37.33%	93.8%	0.46762	0.811	1.468
78.7971698113208	61.11%	84.54%	36.91%	93.62%	0.45651	0.811	1.457
79.2757660167131	61.11%	84.7%	37.16%	93.64%	0.45815	0.811	1.458
79.572192513369	60%	84.7%	36.73%	93.47%	0.44704	0.811	1.447
79.7668997668998	58.89%	84.7%	36.3%	93.3%	0.43593	0.811	1.436
79.7694524495677	58.89%	84.87%	36.55%	93.31%	0.43757	0.811	1.438
80.8067940552017	57.78%	84.87%	36.11%	93.14%	0.42646	0.811	1.426
81.288056206089	57.78%	85.03%	36.36%	93.15%	0.42811	0.811	1.428
81.3165266106442	57.78%	85.2%	36.62%	93.17%	0.42975	0.811	1.430
81.9128329297821	57.78%	85.36%	36.88%	93.18%	0.43140	0.811	1.431
82.0963172804533	56.67%	85.36%	36.43%	93.01%	0.42029	0.811	1.420
83.447619047619	56.67%	85.53%	36.69%	93.02%	0.42193	0.811	1.422
84.4213973799127	55.56%	85.53%	36.23%	92.86%	0.41082	0.811	1.411
84.7912087912088	55.56%	85.69%	36.5%	92.87%	0.41246	0.811	1.412
85.0117647058824	55.56%	85.86%	36.76%	92.88%	0.41411	0.811	1.414
85.5591054313099	55.56%	86.02%	37.04%	92.9%	0.41575	0.811	1.416
85.6132075471698	55.56%	86.18%	37.31%	92.91%	0.41740	0.811	1.417
85.8125	54.44%	86.18%	36.84%	92.74%	0.40629	0.811	1.406
86.1463414634146	54.44%	86.35%	37.12%	92.76%	0.40793	0.811	1.408
86.9373549883991	54.44%	86.51%	37.4%	92.77%	0.40958	0.811	1.410
87.89592760181	54.44%	86.68%	37.69%	92.78%	0.41122	0.811	1.411
88.6849315068493	54.44%	86.84%	37.98%	92.79%	0.41287	0.811	1.413
90.2605210420842	54.44%	87.01%	38.28%	92.81%	0.41451	0.811	1.415
90.4471544715447	54.44%	87.17%	38.58%	92.82%	0.41615	0.811	1.416
90.9862385321101	53.33%	87.17%	38.1%	92.66%	0.40504	0.811	1.405
91.6032608695652	53.33%	87.34%	38.4%	92.67%	0.40669	0.811	1.407
91.9064748201439	52.22%	87.34%	37.9%	92.51%	0.39558	0.811	1.396
92.2222222222222	52.22%	87.5%	38.21%	92.52%	0.39722	0.811	1.397
92.7472527472527	52.22%	87.66%	38.52%	92.53%	0.39887	0.811	1.399
93.1034482758621	52.22%	87.83%	38.84%	92.55%	0.40051	0.811	1.401
93.8055555555556	52.22%	87.99%	39.17%	92.56%	0.40216	0.811	1.402
93.9364303178484	52.22%	88.16%	39.5%	92.57%	0.40380	0.811	1.404

Scale: D-DİMER/PCO2

94.7322970639033	51.11%	88.16%	38.98%	92.41%	0.39269	0.811	1.393
94.7552447552447	51.11%	88.32%	39.32%	92.43%	0.39433	0.811	1.394
96.5194805194805	50%	88.32%	38.79%	92.27%	0.38322	0.811	1.383
96.5324384787472	48.89%	88.32%	38.26%	92.11%	0.37211	0.811	1.372
96.7605633802817	48.89%	88.49%	38.6%	92.12%	0.37376	0.811	1.374
98.8712522045855	48.89%	88.65%	38.94%	92.14%	0.37540	0.811	1.375
99.5907928388747	48.89%	88.82%	39.29%	92.15%	0.37705	0.811	1.377
100.356294536817	48.89%	88.98%	39.64%	92.16%	0.37869	0.811	1.379
101.525423728814	48.89%	89.14%	40%	92.18%	0.38034	0.811	1.380
101.526717557252	48.89%	89.31%	40.37%	92.19%	0.38198	0.811	1.382
102.463768115942	47.78%	89.31%	39.81%	92.03%	0.37087	0.811	1.371
103.070707070707	47.78%	89.47%	40.19%	92.05%	0.37251	0.811	1.373
104.790286975717	47.78%	89.64%	40.57%	92.06%	0.37416	0.811	1.374
105.911330049261	47.78%	89.8%	40.95%	92.07%	0.37580	0.811	1.376
106.88829787234	47.78%	89.97%	41.35%	92.09%	0.37745	0.811	1.377
108.141135972461	47.78%	90.13%	41.75%	92.1%	0.37909	0.811	1.379
110.531914893617	47.78%	90.3%	42.16%	92.11%	0.38074	0.811	1.381
113.324468085106	46.67%	90.3%	41.58%	91.96%	0.36963	0.811	1.370
113.353293413174	45.56%	90.3%	41%	91.81%	0.35852	0.811	1.359
113.895705521472	45.56%	90.46%	41.41%	91.82%	0.36016	0.811	1.360
114.093264248705	44.44%	90.46%	40.82%	91.67%	0.34905	0.811	1.349
114.425287356322	44.44%	90.62%	41.24%	91.68%	0.35069	0.811	1.351
115.447368421053	44.44%	90.79%	41.67%	91.69%	0.35234	0.811	1.352
115.851648351648	44.44%	90.95%	42.11%	91.71%	0.35398	0.811	1.354
116.619318181818	43.33%	90.95%	41.49%	91.56%	0.34287	0.811	1.343
117.251908396947	42.22%	90.95%	40.86%	91.4%	0.33176	0.811	1.332
119.700460829493	42.22%	91.12%	41.3%	91.42%	0.33341	0.811	1.333
122.613065326633	42.22%	91.28%	41.76%	91.43%	0.33505	0.811	1.335
123.615635179153	42.22%	91.45%	42.22%	91.45%	0.33670	0.811	1.337
124.349112426036	42.22%	91.61%	42.7%	91.46%	0.33834	0.811	1.338
125.961123110151	42.22%	91.78%	43.18%	91.48%	0.33999	0.811	1.340
128.903743315508	42.22%	91.94%	43.68%	91.49%	0.34163	0.811	1.342
129.076433121019	42.22%	92.11%	44.19%	91.5%	0.34327	0.811	1.343
130.049751243781	42.22%	92.27%	44.71%	91.52%	0.34492	0.811	1.345
131.211267605634	41.11%	92.27%	44.05%	91.37%	0.33381	0.811	1.334
132.196531791908	41.11%	92.43%	44.58%	91.38%	0.33545	0.811	1.335
132.682403433476	41.11%	92.6%	45.12%	91.4%	0.33710	0.811	1.337
133.302961275626	41.11%	92.76%	45.68%	91.41%	0.33874	0.811	1.339
134.168797953964	40%	92.76%	45%	91.26%	0.32763	0.811	1.328
134.313099041534	40%	92.93%	45.57%	91.28%	0.32928	0.811	1.329
137.492537313433	40%	93.09%	46.15%	91.29%	0.33092	0.811	1.331
139.121447028424	40%	93.26%	46.75%	91.3%	0.33257	0.811	1.333
140.455486542443	40%	93.42%	47.37%	91.32%	0.33421	0.811	1.334
140.758928571429	40%	93.59%	48%	91.33%	0.33586	0.811	1.336
141.78674351585	38.89%	93.59%	47.3%	91.19%	0.32474	0.811	1.325
145.1197053407	37.78%	93.59%	46.58%	91.04%	0.31363	0.811	1.314
147.328244274809	37.78%	93.75%	47.22%	91.05%	0.31528	0.811	1.315
147.507886435331	37.78%	93.91%	47.89%	91.07%	0.31692	0.811	1.317
147.521865889213	37.78%	94.08%	48.57%	91.08%	0.31857	0.811	1.319
149.94623655914	36.67%	94.08%	47.83%	90.94%	0.30746	0.811	1.307
150.182291666667	36.67%	94.24%	48.53%	90.95%	0.30910	0.811	1.309
150.371352785146	35.56%	94.24%	47.76%	90.81%	0.29799	0.811	1.298
152.307692307692	35.56%	94.41%	48.48%	90.82%	0.29963	0.811	1.300

Scale: D-DİMER/PCO2

156.06648199446	34.44%	94.41%	47.69%	90.68%	0.28852	0.811	1.289
158.974358974359	33.33%	94.41%	46.88%	90.54%	0.27741	0.811	1.277
161.349693251534	33.33%	94.57%	47.62%	90.55%	0.27906	0.811	1.279
161.743869209809	33.33%	94.74%	48.39%	90.57%	0.28070	0.811	1.281
162.409638554217	32.22%	94.74%	47.54%	90.42%	0.26959	0.811	1.270
164.642857142857	32.22%	94.9%	48.33%	90.44%	0.27124	0.811	1.271
165.454545454545	32.22%	95.07%	49.15%	90.45%	0.27288	0.811	1.273
165.46485260771	32.22%	95.23%	50%	90.47%	0.27452	0.811	1.275
166.478873239437	31.11%	95.23%	49.12%	90.33%	0.26341	0.811	1.263
168.399071925754	30%	95.23%	48.21%	90.19%	0.25230	0.811	1.252
169.707446808511	30%	95.39%	49.09%	90.2%	0.25395	0.811	1.254
171.701149425287	30%	95.56%	50%	90.22%	0.25559	0.811	1.256
173.032967032967	30%	95.72%	50.94%	90.23%	0.25724	0.811	1.257
174.148351648352	28.89%	95.72%	50%	90.09%	0.24613	0.811	1.246
182.527173913043	28.89%	95.89%	50.98%	90.11%	0.24777	0.811	1.248
183.574879227053	28.89%	96.05%	52%	90.12%	0.24942	0.811	1.249
191.800486618005	28.89%	96.22%	53.06%	90.14%	0.25106	0.811	1.251
193.341346153846	28.89%	96.38%	54.17%	90.15%	0.25270	0.811	1.253
195.291005291005	27.78%	96.38%	53.19%	90.02%	0.24159	0.811	1.242
195.606694560669	27.78%	96.55%	54.35%	90.03%	0.24324	0.811	1.243
199.212827988338	27.78%	96.71%	55.56%	90.05%	0.24488	0.811	1.245
201.845386533666	27.78%	96.88%	56.82%	90.06%	0.24653	0.811	1.247
201.990171990172	27.78%	97.04%	58.14%	90.08%	0.24817	0.811	1.248
204.136690647482	26.67%	97.04%	57.14%	89.94%	0.23706	0.811	1.237
205.045317220544	26.67%	97.2%	58.54%	89.95%	0.23871	0.811	1.239
209.942028985507	26.67%	97.37%	60%	89.97%	0.24035	0.811	1.240
210.33950617284	25.56%	97.37%	58.97%	89.83%	0.22924	0.811	1.229
215.955334987593	25.56%	97.53%	60.53%	89.85%	0.23088	0.811	1.231
219.812030075188	25.56%	97.7%	62.16%	89.86%	0.23253	0.811	1.233
220.05	25.56%	97.86%	63.89%	89.88%	0.23417	0.811	1.234
221.967213114754	25.56%	98.03%	65.71%	89.89%	0.23582	0.811	1.236
224.493243243243	25.56%	98.19%	67.65%	89.91%	0.23746	0.811	1.237
226.643598615917	24.44%	98.19%	66.67%	89.77%	0.22635	0.811	1.226
230.58282208589	23.33%	98.19%	65.62%	89.64%	0.21524	0.811	1.215
234.134275618375	22.22%	98.19%	64.52%	89.51%	0.20413	0.811	1.204
235.514705882353	22.22%	98.36%	66.67%	89.52%	0.20577	0.811	1.206
235.688073394495	22.22%	98.52%	68.97%	89.54%	0.20742	0.811	1.207
236.875	22.22%	98.68%	71.43%	89.55%	0.20906	0.811	1.209
258.436578171091	22.22%	98.85%	74.07%	89.57%	0.21071	0.811	1.211
262.27	22.22%	99.01%	76.92%	89.58%	0.21235	0.811	1.212
265.265017667845	21.11%	99.01%	76%	89.45%	0.20124	0.811	1.201
291.596330275229	20%	99.01%	75%	89.32%	0.19013	0.811	1.190
329.133663366337	18.89%	99.01%	73.91%	89.19%	0.17902	0.811	1.179
376.871035940803	17.78%	99.01%	72.73%	89.05%	0.16791	0.811	1.168
402.204610951009	16.67%	99.01%	71.43%	88.92%	0.15680	0.811	1.157
409.673024523161	15.56%	99.01%	70%	88.79%	0.14569	0.811	1.146
419.906103286385	15.56%	99.18%	73.68%	88.81%	0.14733	0.811	1.147
430.063965884861	15.56%	99.34%	77.78%	88.82%	0.14898	0.811	1.149
455.889724310777	14.44%	99.34%	76.47%	88.69%	0.13787	0.811	1.138
464.649859943978	13.33%	99.34%	75%	88.56%	0.12675	0.811	1.127
464.742268041237	12.22%	99.34%	73.33%	88.43%	0.11564	0.811	1.116
490.695187165775	11.11%	99.34%	71.43%	88.3%	0.10453	0.811	1.105
495.496828752643	10%	99.34%	69.23%	88.18%	0.09342	0.811	1.093

Scale: D-DİMER/PCO2

522.68221574344	10%	99.51%	75%	88.19%	0.09507	0.811	1.095
576.626016260163	8.89%	99.51%	72.73%	88.06%	0.08395	0.811	1.084
577.643312101911	7.78%	99.51%	70%	87.94%	0.07284	0.811	1.073
672.87037037037	6.67%	99.51%	66.67%	87.81%	0.06173	0.811	1.062
699.966555183947	5.56%	99.51%	62.5%	87.68%	0.05062	0.811	1.051
719.651162790698	4.44%	99.51%	57.14%	87.55%	0.03951	0.811	1.040
805.448504983389	3.33%	99.51%	50%	87.43%	0.02840	0.811	1.028
864.97461928934	2.22%	99.51%	40%	87.3%	0.01729	0.811	1.017
938.219696969697	1.11%	99.51%	25%	87.18%	0.00618	0.811	1.006
1149.74564926372	0%	99.51%	0%	87.05%	-0.00493	0.811	0.995
1240.36303630363	0%	99.67%	0%	87.07%	-0.00329	0.811	0.997
2615.46712802768	0%	99.84%	0%	87.09%	-0.00164	0.811	0.998
