# Supplementary material for: Pre-CT risk stratification using the D-dimer/pCO₂ ratio in D-dimer–positive emergency department patients: diagnostic accuracy study
Source: BMC Emerg Med. 2025 Nov 17;25:237. doi: 10.1186/s12873-025-01395-6 (PMC12625727; doi:10.1186/s12873-025-01395-6)
Supplement: Supplementary file 2 — Supplementary Material 2 [file 12873_2025_1395_MOESM2_ESM.pdf]

Scale: D-DİMER/PCO2

Cutpoint	Sensitivity (%)	Specificity (%)	PPV (%)	NPV (%)	Youden's index	AUC	Metric Score
10.8199356913183	100%	0%	4.15%	NaN%	0.00000	0.938	1.000
11.2199036918138	100%	0.15%	4.16%	100%	0.00149	0.938	1.001
12.1098265895954	100%	0.3%	4.17%	100%	0.00299	0.938	1.003
12.2799097065463	100%	0.45%	4.17%	100%	0.00448	0.938	1.004
12.5263157894737	100%	0.6%	4.18%	100%	0.00598	0.938	1.006
12.636815920398	100%	0.75%	4.18%	100%	0.00747	0.938	1.007
12.8856624319419	100%	0.9%	4.19%	100%	0.00897	0.938	1.009
12.957264957265	100%	1.05%	4.2%	100%	0.01046	0.938	1.010
13.1027253668763	100%	1.2%	4.2%	100%	0.01196	0.938	1.012
13.1304347826087	100%	1.35%	4.21%	100%	0.01345	0.938	1.013
13.1639722863741	100%	1.49%	4.22%	100%	0.01495	0.938	1.015
13.1764705882353	100%	1.64%	4.22%	100%	0.01644	0.938	1.016
13.2112068965517	100%	1.79%	4.23%	100%	0.01794	0.938	1.018
13.4122287968442	100%	1.94%	4.23%	100%	0.01943	0.938	1.019
13.4252873563218	100%	2.09%	4.24%	100%	0.02093	0.938	1.021
13.444976076555	100%	2.24%	4.25%	100%	0.02242	0.938	1.022
13.5261194029851	100%	2.39%	4.25%	100%	0.02392	0.938	1.024
13.5322195704057	100%	2.54%	4.26%	100%	0.02541	0.938	1.025
13.6623376623377	100%	2.69%	4.26%	100%	0.02691	0.938	1.027
13.6725663716814	100%	2.84%	4.27%	100%	0.02840	0.938	1.028
13.7250554323725	100%	2.99%	4.28%	100%	0.02990	0.938	1.030
13.7391304347826	100%	3.14%	4.28%	100%	0.03139	0.938	1.031
13.8796680497925	100%	3.29%	4.29%	100%	0.03288	0.938	1.033
14.1176470588235	100%	3.44%	4.3%	100%	0.03438	0.938	1.034
14.1188524590164	100%	3.59%	4.3%	100%	0.03587	0.938	1.036
14.1794871794872	100%	3.74%	4.31%	100%	0.03737	0.938	1.037
14.2154566744731	100%	3.89%	4.32%	100%	0.03886	0.938	1.039
14.35960591133	100%	4.04%	4.32%	100%	0.04036	0.938	1.040
14.3914081145585	100%	4.19%	4.33%	100%	0.04185	0.938	1.042
14.572864321608	100%	4.33%	4.33%	100%	0.04335	0.938	1.043
14.8314606741573	100%	4.48%	4.34%	100%	0.04484	0.938	1.045
15.1304347826087	100%	4.63%	4.35%	100%	0.04634	0.938	1.046
15.4661016949153	100%	4.78%	4.35%	100%	0.04783	0.938	1.048
15.5741127348643	100%	4.93%	4.36%	100%	0.04933	0.938	1.049
15.6105610561056	100%	5.08%	4.37%	100%	0.05082	0.938	1.051
15.6315789473684	100%	5.23%	4.37%	100%	0.05232	0.938	1.052
15.692007797271	100%	5.38%	4.38%	100%	0.05381	0.938	1.054
15.8049886621315	100%	5.53%	4.39%	100%	0.05531	0.938	1.055
15.9061833688699	100%	5.68%	4.39%	100%	0.05680	0.938	1.057
15.9512195121951	100%	5.83%	4.4%	100%	0.05830	0.938	1.058
16.0084033613445	100%	5.98%	4.41%	100%	0.05979	0.938	1.060
16.0263157894737	100%	6.13%	4.41%	100%	0.06129	0.938	1.061
16.0483870967742	100%	6.28%	4.42%	100%	0.06278	0.938	1.063
16.0952380952381	100%	6.43%	4.43%	100%	0.06428	0.938	1.064
16.0986547085202	100%	6.58%	4.43%	100%	0.06577	0.938	1.066
16.1282051282051	100%	6.73%	4.44%	100%	0.06726	0.938	1.067
16.1662198391421	100%	6.88%	4.45%	100%	0.06876	0.938	1.069
16.2781954887218	100%	7.03%	4.45%	100%	0.07025	0.938	1.070
16.3466042154567	100%	7.17%	4.46%	100%	0.07175	0.938	1.072
16.4364640883978	100%	7.32%	4.47%	100%	0.07324	0.938	1.073

16.474358974359	100%	7.47%	4.48%	100%	0.07474	0.938	1.075
16.7331670822943	100%	7.62%	4.48%	100%	0.07623	0.938	1.076
16.7337807606264	100%	7.77%	4.49%	100%	0.07773	0.938	1.078
16.8181818181818	100%	7.92%	4.5%	100%	0.07922	0.938	1.079
16.8241469816273	100%	8.07%	4.5%	100%	0.08072	0.938	1.081
16.9248291571754	100%	8.22%	4.51%	100%	0.08221	0.938	1.082
16.9298245614035	100%	8.37%	4.52%	100%	0.08371	0.938	1.084
16.9402985074627	100%	8.52%	4.52%	100%	0.08520	0.938	1.085
16.9594594594595	100%	8.67%	4.53%	100%	0.08670	0.938	1.087
16.991643454039	100%	8.82%	4.54%	100%	0.08819	0.938	1.088
17.1867007672634	100%	8.97%	4.55%	100%	0.08969	0.938	1.090
17.2	100%	9.12%	4.55%	100%	0.09118	0.938	1.091
17.20823798627	100%	9.27%	4.56%	100%	0.09268	0.938	1.093
17.2549019607843	100%	9.42%	4.57%	100%	0.09417	0.938	1.094
17.2727272727273	100%	9.57%	4.57%	100%	0.09567	0.938	1.096
17.2986577181208	100%	9.72%	4.58%	100%	0.09716	0.938	1.097
17.34	100%	9.87%	4.59%	100%	0.09865	0.938	1.099
17.4285714285714	100%	10.01%	4.6%	100%	0.10015	0.938	1.100
17.4298056155508	100%	10.16%	4.6%	100%	0.10164	0.938	1.102
17.4352331606218	100%	10.31%	4.61%	100%	0.10314	0.938	1.103
17.4678111587983	100%	10.46%	4.62%	100%	0.10463	0.938	1.105
17.5	100%	10.61%	4.63%	100%	0.10613	0.938	1.106
17.5211267605634	100%	10.76%	4.63%	100%	0.10762	0.938	1.108
17.5510204081633	100%	10.91%	4.64%	100%	0.10912	0.938	1.109
17.6381909547739	100%	11.06%	4.65%	100%	0.11061	0.938	1.111
17.6437847866419	100%	11.21%	4.65%	100%	0.11211	0.938	1.112
17.6701570680628	100%	11.36%	4.66%	100%	0.11360	0.938	1.114
17.6781609195402	100%	11.51%	4.67%	100%	0.11510	0.938	1.115
17.7142857142857	100%	11.66%	4.68%	100%	0.11659	0.938	1.117
17.7530864197531	100%	11.81%	4.68%	100%	0.11809	0.938	1.118
17.787610619469	100%	11.96%	4.69%	100%	0.11958	0.938	1.120
17.8181818181818	100%	12.11%	4.7%	100%	0.12108	0.938	1.121
17.841726618705	100%	12.26%	4.71%	100%	0.12257	0.938	1.123
17.8961038961039	100%	12.41%	4.72%	100%	0.12407	0.938	1.124
17.921875	100%	12.56%	4.72%	100%	0.12556	0.938	1.126
17.9820627802691	100%	12.71%	4.73%	100%	0.12706	0.938	1.127
17.9879275653924	100%	12.86%	4.74%	100%	0.12855	0.938	1.129
17.9901960784314	100%	13%	4.75%	100%	0.13004	0.938	1.130
18.0449826989619	100%	13.15%	4.75%	100%	0.13154	0.938	1.132
18.0875202593193	100%	13.3%	4.76%	100%	0.13303	0.938	1.133
18.089552238806	100%	13.45%	4.77%	100%	0.13453	0.938	1.135
18.10888252149	100%	13.6%	4.78%	100%	0.13602	0.938	1.136
18.141592920354	100%	13.75%	4.79%	100%	0.13752	0.938	1.138
18.1497797356828	100%	13.9%	4.79%	100%	0.13901	0.938	1.139
18.4257206208426	100%	14.05%	4.8%	100%	0.14051	0.938	1.141
18.4517766497462	100%	14.2%	4.81%	100%	0.14200	0.938	1.142
18.452380952381	100%	14.35%	4.82%	100%	0.14350	0.938	1.143
18.5416666666667	100%	14.5%	4.83%	100%	0.14499	0.938	1.145
18.5539714867617	100%	14.65%	4.83%	100%	0.14649	0.938	1.146
18.5552407932011	100%	14.8%	4.84%	100%	0.14798	0.938	1.148
18.5828877005348	100%	14.95%	4.85%	100%	0.14948	0.938	1.149
18.7117903930131	100%	15.1%	4.86%	100%	0.15097	0.938	1.151
18.716049382716	100%	15.25%	4.87%	100%	0.15247	0.938	1.152

18.780487804878	100%	15.4%	4.87%	100%	0.15396	0.938	1.154
18.8183807439825	100%	15.55%	4.88%	100%	0.15546	0.938	1.155
18.8235294117647	100%	15.7%	4.89%	100%	0.15695	0.938	1.157
18.8612836438923	100%	15.84%	4.9%	100%	0.15845	0.938	1.158
18.8640973630832	100%	15.99%	4.91%	100%	0.15994	0.938	1.160
18.9592760180995	100%	16.14%	4.92%	100%	0.16143	0.938	1.161
18.9830508474576	100%	16.29%	4.92%	100%	0.16293	0.938	1.163
19.0184049079755	100%	16.44%	4.93%	100%	0.16442	0.938	1.164
19.0439770554493	100%	16.59%	4.94%	100%	0.16592	0.938	1.166
19.0724637681159	100%	16.74%	4.95%	100%	0.16741	0.938	1.167
19.0818858560794	100%	16.89%	4.96%	100%	0.16891	0.938	1.169
19.1085271317829	100%	17.04%	4.97%	100%	0.17040	0.938	1.170
19.1947565543071	100%	17.19%	4.97%	100%	0.17190	0.938	1.172
19.2050209205021	100%	17.34%	4.98%	100%	0.17339	0.938	1.173
19.3565683646113	100%	17.49%	4.99%	100%	0.17489	0.938	1.175
19.4019933554817	100%	17.64%	5%	100%	0.17638	0.938	1.176
19.4085027726433	100%	17.79%	5.01%	100%	0.17788	0.938	1.178
19.478021978022	100%	17.94%	5.02%	100%	0.17937	0.938	1.179
19.5032397408207	100%	18.09%	5.03%	100%	0.18087	0.938	1.181
19.504132231405	100%	18.24%	5.03%	100%	0.18236	0.938	1.182
19.5287958115183	100%	18.39%	5.04%	100%	0.18386	0.938	1.184
19.5377128953771	100%	18.54%	5.05%	100%	0.18535	0.938	1.185
19.6567505720824	100%	18.68%	5.06%	100%	0.18685	0.938	1.187
19.6626506024096	100%	18.83%	5.07%	100%	0.18834	0.938	1.188
19.7272727272727	100%	18.98%	5.08%	100%	0.18984	0.938	1.190
19.7820163487738	100%	19.13%	5.09%	100%	0.19133	0.938	1.191
19.8547215496368	100%	19.28%	5.1%	100%	0.19283	0.938	1.193
19.855421686747	100%	19.43%	5.11%	100%	0.19432	0.938	1.194
19.8606271777003	100%	19.58%	5.11%	100%	0.19581	0.938	1.196
19.9058380414313	100%	19.73%	5.12%	100%	0.19731	0.938	1.197
19.9698795180723	100%	19.88%	5.13%	100%	0.19880	0.938	1.199
20.1663893510815	100%	20.03%	5.14%	100%	0.20030	0.938	1.200
20.3287671232877	100%	20.18%	5.15%	100%	0.20179	0.938	1.202
20.3303303303303	100%	20.33%	5.16%	100%	0.20329	0.938	1.203
20.3703703703704	100%	20.48%	5.17%	100%	0.20478	0.938	1.205
20.4455445544554	100%	20.63%	5.18%	100%	0.20628	0.938	1.206
20.455764075067	100%	20.78%	5.19%	100%	0.20777	0.938	1.208
20.6306306306306	100%	20.93%	5.2%	100%	0.20927	0.938	1.209
20.7179487179487	100%	21.08%	5.21%	100%	0.21076	0.938	1.211
20.7266982622433	100%	21.23%	5.22%	100%	0.21226	0.938	1.212
20.7564575645756	100%	21.38%	5.23%	100%	0.21375	0.938	1.214
20.8478802992519	100%	21.52%	5.23%	100%	0.21525	0.938	1.215
20.8727272727273	100%	21.67%	5.24%	100%	0.21674	0.938	1.217
20.961145194274	100%	21.82%	5.25%	100%	0.21824	0.938	1.218
21.039755351682	100%	21.97%	5.26%	100%	0.21973	0.938	1.220
21.0765550239234	100%	22.12%	5.27%	100%	0.22123	0.938	1.221
21.2476722532588	100%	22.27%	5.28%	100%	0.22272	0.938	1.223
21.2605042016807	100%	22.42%	5.29%	100%	0.22422	0.938	1.224
21.2621359223301	100%	22.57%	5.3%	100%	0.22571	0.938	1.226
21.3380281690141	100%	22.72%	5.31%	100%	0.22720	0.938	1.227
21.3432835820896	100%	22.87%	5.32%	100%	0.22870	0.938	1.229
21.3532110091743	100%	23.02%	5.33%	100%	0.23019	0.938	1.230
21.3970588235294	100%	23.17%	5.34%	100%	0.23169	0.938	1.232

Scale: D-DİMER/PCO2

21.448275862069	100%	23.32%	5.35%	100%	0.23318	0.938	1.233
21.6071428571429	100%	23.47%	5.36%	100%	0.23468	0.938	1.235
21.6858237547893	100%	23.62%	5.37%	100%	0.23617	0.938	1.236
21.7391304347826	100%	23.77%	5.38%	100%	0.23767	0.938	1.238
21.7535545023697	100%	23.92%	5.39%	100%	0.23916	0.938	1.239
21.7811704834606	100%	24.07%	5.4%	100%	0.24066	0.938	1.241
21.8924731182796	100%	24.22%	5.41%	100%	0.24215	0.938	1.242
22.0815450643777	100%	24.36%	5.42%	100%	0.24365	0.938	1.244
22.0954907161804	100%	24.51%	5.43%	100%	0.24514	0.938	1.245
22.1292775665399	100%	24.66%	5.44%	100%	0.24664	0.938	1.247
22.2168674698795	100%	24.81%	5.45%	100%	0.24813	0.938	1.248
22.2356495468278	100%	24.96%	5.46%	100%	0.24963	0.938	1.250
22.2795698924731	100%	25.11%	5.47%	100%	0.25112	0.938	1.251
22.3062730627306	100%	25.26%	5.48%	100%	0.25262	0.938	1.253
22.375	100%	25.41%	5.49%	100%	0.25411	0.938	1.254
22.4220183486239	100%	25.56%	5.5%	100%	0.25561	0.938	1.256
22.4333333333333	100%	25.71%	5.51%	100%	0.25710	0.938	1.257
22.6588235294118	100%	25.86%	5.52%	100%	0.25859	0.938	1.259
22.8238341968912	100%	26.01%	5.53%	100%	0.26009	0.938	1.260
22.8482972136223	100%	26.16%	5.54%	100%	0.26158	0.938	1.262
22.8883495145631	100%	26.31%	5.56%	100%	0.26308	0.938	1.263
22.9032258064516	100%	26.46%	5.57%	100%	0.26457	0.938	1.265
22.903981264637	100%	26.61%	5.58%	100%	0.26607	0.938	1.266
22.9339853300734	100%	26.76%	5.59%	100%	0.26756	0.938	1.268
22.962962962963	100%	26.91%	5.6%	100%	0.26906	0.938	1.269
23.0618892508143	100%	27.06%	5.61%	100%	0.27055	0.938	1.271
23.0740740740741	100%	27.2%	5.62%	100%	0.27205	0.938	1.272
23.1552162849873	100%	27.35%	5.63%	100%	0.27354	0.938	1.274
23.2553191489362	100%	27.5%	5.64%	100%	0.27504	0.938	1.275
23.3407572383074	100%	27.65%	5.65%	100%	0.27653	0.938	1.277
23.3644859813084	100%	27.8%	5.66%	100%	0.27803	0.938	1.278
23.4924078091106	100%	27.95%	5.68%	100%	0.27952	0.938	1.280
23.5714285714286	100%	28.1%	5.69%	100%	0.28102	0.938	1.281
23.5787321063395	100%	28.25%	5.7%	100%	0.28251	0.938	1.283
23.7193763919822	100%	28.4%	5.71%	100%	0.28401	0.938	1.284
23.7264150943396	100%	28.55%	5.72%	100%	0.28550	0.938	1.286
23.7383177570093	100%	28.7%	5.73%	100%	0.28700	0.938	1.287
23.7558685446009	100%	28.85%	5.74%	100%	0.28849	0.938	1.288
23.8235294117647	100%	29%	5.75%	100%	0.28999	0.938	1.290
23.8477366255144	100%	29.15%	5.77%	100%	0.29148	0.938	1.291
23.8548752834467	100%	29.3%	5.78%	100%	0.29297	0.938	1.293
23.9007092198582	100%	29.45%	5.79%	100%	0.29447	0.938	1.294
24.0960451977401	100%	29.6%	5.8%	100%	0.29596	0.938	1.296
24.1114058355438	100%	29.75%	5.81%	100%	0.29746	0.938	1.297
24.2025862068966	100%	29.9%	5.82%	100%	0.29895	0.938	1.299
24.4	100%	30.04%	5.84%	100%	0.30045	0.938	1.300
24.4309927360775	100%	30.19%	5.85%	100%	0.30194	0.938	1.302
24.4487427466151	100%	30.34%	5.86%	100%	0.30344	0.938	1.303
24.4796380090498	100%	30.49%	5.87%	100%	0.30493	0.938	1.305
24.5244956772334	100%	30.64%	5.88%	100%	0.30643	0.938	1.306
24.6578947368421	100%	30.79%	5.89%	100%	0.30792	0.938	1.308
24.688995215311	100%	30.94%	5.91%	100%	0.30942	0.938	1.309
24.7545219638243	100%	31.09%	5.92%	100%	0.31091	0.938	1.311

Scale: D-DİMER/PCO2

24.7674418604651	100%	31.24%	5.93%	100%	0.31241	0.938	1.312
24.8214285714286	100%	31.39%	5.94%	100%	0.31390	0.938	1.314
24.8536585365854	100%	31.54%	5.95%	100%	0.31540	0.938	1.315
24.915611814346	100%	31.69%	5.97%	100%	0.31689	0.938	1.317
24.9175824175824	100%	31.84%	5.98%	100%	0.31839	0.938	1.318
24.9289099526066	100%	31.99%	5.99%	100%	0.31988	0.938	1.320
24.9465240641711	100%	32.14%	6%	100%	0.32138	0.938	1.321
24.965034965035	100%	32.29%	6.02%	100%	0.32287	0.938	1.323
24.9761904761905	100%	32.44%	6.03%	100%	0.32436	0.938	1.324
24.9882352941176	100%	32.59%	6.04%	100%	0.32586	0.938	1.326
25	100%	32.74%	6.05%	100%	0.32735	0.938	1.327
25.0147928994083	100%	32.88%	6.07%	100%	0.32885	0.938	1.329
25.1653944020356	100%	33.03%	6.08%	100%	0.33034	0.938	1.330
25.2300242130751	100%	33.18%	6.09%	100%	0.33184	0.938	1.332
25.4166666666667	100%	33.33%	6.11%	100%	0.33333	0.938	1.333
25.4460093896714	100%	33.48%	6.12%	100%	0.33483	0.938	1.335
25.4741379310345	100%	33.63%	6.13%	100%	0.33632	0.938	1.336
25.531914893617	100%	33.78%	6.14%	100%	0.33782	0.938	1.338
25.5351681957187	100%	33.93%	6.16%	100%	0.33931	0.938	1.339
25.5625	100%	34.08%	6.17%	100%	0.34081	0.938	1.341
25.5690072639225	100%	34.23%	6.18%	100%	0.34230	0.938	1.342
25.5882352941176	100%	34.38%	6.2%	100%	0.34380	0.938	1.344
25.6300268096515	100%	34.53%	6.21%	100%	0.34529	0.938	1.345
25.7824933687003	100%	34.68%	6.22%	100%	0.34679	0.938	1.347
25.800464037123	100%	34.83%	6.24%	100%	0.34828	0.938	1.348
25.8208955223881	100%	34.98%	6.25%	100%	0.34978	0.938	1.350
25.8415841584158	100%	35.13%	6.26%	100%	0.35127	0.938	1.351
25.8595641646489	100%	35.28%	6.28%	100%	0.35277	0.938	1.353
25.8785249457701	100%	35.43%	6.29%	100%	0.35426	0.938	1.354
25.9751037344398	100%	35.58%	6.3%	100%	0.35575	0.938	1.356
26.0043668122271	100%	35.72%	6.32%	100%	0.35725	0.938	1.357
26.0722891566265	100%	35.87%	6.33%	100%	0.35874	0.938	1.359
26.0801781737194	100%	36.02%	6.35%	100%	0.36024	0.938	1.360
26.1096605744125	100%	36.17%	6.36%	100%	0.36173	0.938	1.362
26.1467889908257	100%	36.32%	6.37%	100%	0.36323	0.938	1.363
26.1715481171548	100%	36.47%	6.39%	100%	0.36472	0.938	1.365
26.2368421052632	100%	36.62%	6.4%	100%	0.36622	0.938	1.366
26.2385321100917	100%	36.77%	6.42%	100%	0.36771	0.938	1.368
26.3013698630137	100%	36.92%	6.43%	100%	0.36921	0.938	1.369
26.5029469548134	100%	37.07%	6.44%	100%	0.37070	0.938	1.371
26.5131578947368	100%	37.22%	6.46%	100%	0.37220	0.938	1.372
26.5333333333333	100%	37.37%	6.47%	100%	0.37369	0.938	1.374
26.5860215053763	100%	37.52%	6.49%	100%	0.37519	0.938	1.375
26.6164154103853	100%	37.67%	6.5%	100%	0.37668	0.938	1.377
26.6835443037975	100%	37.82%	6.52%	100%	0.37818	0.938	1.378
26.7037861915368	100%	37.97%	6.53%	100%	0.37967	0.938	1.380
26.9463087248322	100%	38.12%	6.55%	100%	0.38117	0.938	1.381
26.9889502762431	100%	38.27%	6.56%	100%	0.38266	0.938	1.383
27.0676691729323	100%	38.42%	6.58%	100%	0.38416	0.938	1.384
27.1194379391101	100%	38.57%	6.59%	100%	0.38565	0.938	1.386
27.1230158730159	100%	38.71%	6.61%	100%	0.38714	0.938	1.387
27.1276595744681	100%	38.86%	6.62%	100%	0.38864	0.938	1.389
27.1304347826087	100%	39.01%	6.64%	100%	0.39013	0.938	1.390

Scale: D-DİMER/PCO2

27.1363636363636	100%	39.16%	6.65%	100%	0.39163	0.938	1.392
27.2768878718535	100%	39.31%	6.67%	100%	0.39312	0.938	1.393
27.3414634146341	100%	39.46%	6.68%	100%	0.39462	0.938	1.395
27.3655913978495	100%	39.61%	6.7%	100%	0.39611	0.938	1.396
27.3700305810398	100%	39.76%	6.71%	100%	0.39761	0.938	1.398
27.4025974025974	100%	39.91%	6.73%	100%	0.39910	0.938	1.399
27.4260355029586	100%	40.06%	6.74%	100%	0.40060	0.938	1.401
27.43	100%	40.21%	6.76%	100%	0.40209	0.938	1.402
27.5268817204301	100%	40.36%	6.78%	100%	0.40359	0.938	1.404
27.7777777777778	100%	40.51%	6.79%	100%	0.40508	0.938	1.405
27.8228782287823	100%	40.66%	6.81%	100%	0.40658	0.938	1.407
27.8772378516624	100%	40.81%	6.82%	100%	0.40807	0.938	1.408
27.9057591623037	100%	40.96%	6.84%	100%	0.40957	0.938	1.410
27.9190751445087	100%	41.11%	6.86%	100%	0.41106	0.938	1.411
28.1185567010309	100%	41.26%	6.87%	100%	0.41256	0.938	1.413
28.125	100%	41.41%	6.89%	100%	0.41405	0.938	1.414
28.1542056074766	100%	41.55%	6.9%	100%	0.41555	0.938	1.416
28.1571428571429	100%	41.7%	6.92%	100%	0.41704	0.938	1.417
28.3238636363636	100%	41.85%	6.94%	100%	0.41854	0.938	1.419
28.3253588516746	100%	42%	6.95%	100%	0.42003	0.938	1.420
28.4894837476099	100%	42.15%	6.97%	100%	0.42152	0.938	1.422
28.5714285714286	100%	42.3%	6.99%	100%	0.42302	0.938	1.423
28.6545454545455	100%	42.45%	7%	100%	0.42451	0.938	1.425
28.711943793911	100%	42.6%	7.02%	100%	0.42601	0.938	1.426
28.8582677165354	100%	42.75%	7.04%	100%	0.42750	0.938	1.428
28.8642659279778	100%	42.9%	7.06%	100%	0.42900	0.938	1.429
28.9819004524887	100%	43.05%	7.07%	100%	0.43049	0.938	1.430
28.9893617021277	100%	43.2%	7.09%	100%	0.43199	0.938	1.432
29.0461997019374	100%	43.35%	7.11%	100%	0.43348	0.938	1.433
29.0748898678414	100%	43.5%	7.13%	100%	0.43498	0.938	1.435
29.1136363636364	100%	43.65%	7.14%	100%	0.43647	0.938	1.436
29.1150442477876	100%	43.8%	7.16%	100%	0.43797	0.938	1.438
29.1412742382271	100%	43.95%	7.18%	100%	0.43946	0.938	1.439
29.3017456359102	100%	44.1%	7.2%	100%	0.44096	0.938	1.441
29.4858156028369	100%	44.25%	7.21%	100%	0.44245	0.938	1.442
29.5529411764706	100%	44.39%	7.23%	100%	0.44395	0.938	1.444
29.725	100%	44.54%	7.25%	100%	0.44544	0.938	1.445
29.7385620915033	100%	44.69%	7.27%	100%	0.44694	0.938	1.447
29.7527472527473	100%	44.84%	7.29%	100%	0.44843	0.938	1.448
29.7984886649874	100%	44.99%	7.3%	100%	0.44993	0.938	1.450
29.8123324396783	100%	45.14%	7.32%	100%	0.45142	0.938	1.451
29.8257080610022	100%	45.29%	7.34%	100%	0.45291	0.938	1.453
29.9418604651163	100%	45.44%	7.36%	100%	0.45441	0.938	1.454
30.0242718446602	100%	45.59%	7.38%	100%	0.45590	0.938	1.456
30.1814516129032	100%	45.74%	7.4%	100%	0.45740	0.938	1.457
30.183299389002	100%	45.89%	7.42%	100%	0.45889	0.938	1.459
30.3009259259259	100%	46.04%	7.44%	100%	0.46039	0.938	1.460
30.3174603174603	100%	46.19%	7.46%	100%	0.46188	0.938	1.462
30.6	100%	46.34%	7.47%	100%	0.46338	0.938	1.463
30.613810741688	100%	46.49%	7.49%	100%	0.46487	0.938	1.465
30.6474820143885	100%	46.64%	7.51%	100%	0.46637	0.938	1.466
30.7075471698113	100%	46.79%	7.53%	100%	0.46786	0.938	1.468
30.7377049180328	100%	46.94%	7.55%	100%	0.46936	0.938	1.469

Scale: D-DİMER/PCO2

30.7600950118765	100%	47.09%	7.57%	100%	0.47085	0.938	1.471
30.8208955223881	100%	47.23%	7.59%	100%	0.47235	0.938	1.472
30.8396946564886	100%	47.38%	7.61%	100%	0.47384	0.938	1.474
30.873786407767	100%	47.53%	7.63%	100%	0.47534	0.938	1.475
31.1764705882353	100%	47.68%	7.65%	100%	0.47683	0.938	1.477
31.2190082644628	100%	47.83%	7.67%	100%	0.47833	0.938	1.478
31.2389380530973	100%	47.98%	7.69%	100%	0.47982	0.938	1.480
31.3480392156863	100%	48.13%	7.71%	100%	0.48132	0.938	1.481
31.4565826330532	100%	48.28%	7.73%	100%	0.48281	0.938	1.483
31.5667311411992	100%	48.43%	7.75%	100%	0.48430	0.938	1.484
31.5722120658135	100%	48.58%	7.77%	100%	0.48580	0.938	1.486
31.5909090909091	100%	48.73%	7.8%	100%	0.48729	0.938	1.487
31.6754850088183	100%	48.88%	7.82%	100%	0.48879	0.938	1.489
31.6847826086957	100%	49.03%	7.84%	100%	0.49028	0.938	1.490
31.9723183391003	100%	49.18%	7.86%	100%	0.49178	0.938	1.492
31.9954648526077	100%	49.33%	7.88%	100%	0.49327	0.938	1.493
32.1441124780316	100%	49.48%	7.9%	100%	0.49477	0.938	1.495
32.2588235294118	100%	49.63%	7.92%	100%	0.49626	0.938	1.496
32.3770491803279	100%	49.78%	7.95%	100%	0.49776	0.938	1.498
32.4545454545455	100%	49.93%	7.97%	100%	0.49925	0.938	1.499
32.4931506849315	100%	50.07%	7.99%	100%	0.50075	0.938	1.501
32.5770308123249	100%	50.22%	8.01%	100%	0.50224	0.938	1.502
32.5862068965517	100%	50.37%	8.03%	100%	0.50374	0.938	1.504
32.7083333333333	100%	50.52%	8.06%	100%	0.50523	0.938	1.505
32.7710843373494	100%	50.67%	8.08%	100%	0.50673	0.938	1.507
32.7835051546392	100%	50.82%	8.1%	100%	0.50822	0.938	1.508
32.8518518518519	100%	50.97%	8.12%	100%	0.50972	0.938	1.510
33.1111111111111	100%	51.12%	8.15%	100%	0.51121	0.938	1.511
33.2960893854749	100%	51.27%	8.17%	100%	0.51271	0.938	1.513
33.5149863760218	100%	51.42%	8.19%	100%	0.51420	0.938	1.514
33.5421166306695	100%	51.57%	8.22%	100%	0.51570	0.938	1.516
33.5526315789474	100%	51.72%	8.24%	100%	0.51719	0.938	1.517
33.5693215339233	100%	51.87%	8.26%	100%	0.51868	0.938	1.519
33.5772357723577	100%	52.02%	8.29%	100%	0.52018	0.938	1.520
33.7536656891496	100%	52.17%	8.31%	100%	0.52167	0.938	1.522
33.7783375314861	100%	52.32%	8.33%	100%	0.52317	0.938	1.523
33.9800995024876	100%	52.47%	8.36%	100%	0.52466	0.938	1.525
34.0992167101828	100%	52.62%	8.38%	100%	0.52616	0.938	1.526
34.1312056737589	100%	52.77%	8.41%	100%	0.52765	0.938	1.528
34.188790560472	100%	52.91%	8.43%	100%	0.52915	0.938	1.529
34.2105263157895	100%	53.06%	8.45%	100%	0.53064	0.938	1.531
34.2253521126761	100%	53.21%	8.48%	100%	0.53214	0.938	1.532
34.2595978062157	100%	53.36%	8.5%	100%	0.53363	0.938	1.534
34.2857142857143	100%	53.51%	8.53%	100%	0.53513	0.938	1.535
34.4242424242424	100%	53.66%	8.55%	100%	0.53662	0.938	1.537
34.5185185185185	100%	53.81%	8.58%	100%	0.53812	0.938	1.538
34.7529411764706	100%	53.96%	8.61%	100%	0.53961	0.938	1.540
34.8	100%	54.11%	8.63%	100%	0.54111	0.938	1.541
34.8816568047337	100%	54.26%	8.66%	100%	0.54260	0.938	1.543
34.9049429657795	100%	54.41%	8.68%	100%	0.54410	0.938	1.544
34.9063670411985	100%	54.56%	8.71%	100%	0.54559	0.938	1.546
35.0928381962865	100%	54.71%	8.73%	100%	0.54709	0.938	1.547
35.2823920265781	100%	54.86%	8.76%	100%	0.54858	0.938	1.549

35.6119402985075	100%	55.01%	8.79%	100%	0.55007	0.938	1.550
35.8287795992714	100%	55.16%	8.81%	100%	0.55157	0.938	1.552
35.8659217877095	100%	55.31%	8.84%	100%	0.55306	0.938	1.553
35.9910913140312	100%	55.46%	8.87%	100%	0.55456	0.938	1.555
36.0722891566265	100%	55.61%	8.9%	100%	0.55605	0.938	1.556
36.25	100%	55.75%	8.92%	100%	0.55755	0.938	1.558
36.2531017369727	100%	55.9%	8.95%	100%	0.55904	0.938	1.559
36.2569832402235	100%	56.05%	8.98%	100%	0.56054	0.938	1.561
36.3488372093023	100%	56.2%	9.01%	100%	0.56203	0.938	1.562
36.3982102908277	100%	56.35%	9.03%	100%	0.56353	0.938	1.564
36.4110429447853	100%	56.5%	9.06%	100%	0.56502	0.938	1.565
36.4353312302839	100%	56.65%	9.09%	100%	0.56652	0.938	1.567
36.4490861618799	100%	56.8%	9.12%	100%	0.56801	0.938	1.568
36.5507246376812	100%	56.95%	9.15%	100%	0.56951	0.938	1.570
36.7025862068966	100%	57.1%	9.18%	100%	0.57100	0.938	1.571
36.8238993710692	100%	57.25%	9.21%	100%	0.57250	0.938	1.572
36.916890080429	100%	57.4%	9.24%	100%	0.57399	0.938	1.574
37	100%	57.55%	9.27%	100%	0.57549	0.938	1.575
37.2378516624041	100%	57.7%	9.29%	100%	0.57698	0.938	1.577
37.3233404710921	100%	57.85%	9.32%	100%	0.57848	0.938	1.578
37.4055415617128	100%	58%	9.35%	100%	0.57997	0.938	1.580
37.488038277512	100%	58.15%	9.39%	100%	0.58146	0.938	1.581
37.5466666666667	100%	58.3%	9.42%	100%	0.58296	0.938	1.583
37.6599063962559	100%	58.45%	9.45%	100%	0.58445	0.938	1.584
37.7941176470588	100%	58.59%	9.48%	100%	0.58595	0.938	1.586
38	100%	58.74%	9.51%	100%	0.58744	0.938	1.587
38.1774580335731	100%	58.89%	9.54%	100%	0.58894	0.938	1.589
38.2920792079208	100%	59.04%	9.57%	100%	0.59043	0.938	1.590
38.34	100%	59.19%	9.6%	100%	0.59193	0.938	1.592
38.3555555555556	100%	59.34%	9.63%	100%	0.59342	0.938	1.593
38.4275184275184	100%	59.49%	9.67%	100%	0.59492	0.938	1.595
38.4600760456274	100%	59.64%	9.7%	100%	0.59641	0.938	1.596
38.54	100%	59.79%	9.73%	100%	0.59791	0.938	1.598
38.6453201970443	100%	59.94%	9.76%	100%	0.59940	0.938	1.599
38.6567164179104	100%	60.09%	9.8%	100%	0.60090	0.938	1.601
38.75	100%	60.24%	9.83%	100%	0.60239	0.938	1.602
38.7719298245614	100%	60.39%	9.86%	100%	0.60389	0.938	1.604
39.0361445783133	100%	60.54%	9.9%	100%	0.60538	0.938	1.605
39.0728476821192	100%	60.69%	9.93%	100%	0.60688	0.938	1.607
39.08	100%	60.84%	9.97%	100%	0.60837	0.938	1.608
39.1002570694087	100%	60.99%	10%	100%	0.60987	0.938	1.610
39.125	100%	61.14%	10.03%	100%	0.61136	0.938	1.611
39.5043731778426	100%	61.29%	10.07%	100%	0.61286	0.938	1.613
40.3783783783784	100%	61.43%	10.1%	100%	0.61435	0.938	1.614
40.7142857142857	100%	61.58%	10.14%	100%	0.61584	0.938	1.616
40.8219178082192	100%	61.73%	10.18%	100%	0.61734	0.938	1.617
41.020942408377	100%	61.88%	10.21%	100%	0.61883	0.938	1.619
41.0334346504559	100%	62.03%	10.25%	100%	0.62033	0.938	1.620
41.5222482435597	100%	62.18%	10.28%	100%	0.62182	0.938	1.622
41.7204301075269	100%	62.33%	10.32%	100%	0.62332	0.938	1.623
41.8588235294118	100%	62.48%	10.36%	100%	0.62481	0.938	1.625
41.9315403422983	100%	62.63%	10.39%	100%	0.62631	0.938	1.626
41.9402985074627	100%	62.78%	10.43%	100%	0.62780	0.938	1.628

41.9714964370546	100%	62.93%	10.47%	100%	0.62930	0.938	1.629
42.0689655172414	100%	63.08%	10.51%	100%	0.63079	0.938	1.631
42.1375921375921	100%	63.23%	10.55%	100%	0.63229	0.938	1.632
42.1739130434783	100%	63.38%	10.58%	100%	0.63378	0.938	1.634
42.2796352583587	100%	63.53%	10.62%	100%	0.63528	0.938	1.635
42.3512747875354	100%	63.68%	10.66%	100%	0.63677	0.938	1.637
42.434554973822	100%	63.83%	10.7%	100%	0.63827	0.938	1.638
42.5449101796407	100%	63.98%	10.74%	100%	0.63976	0.938	1.640
42.5566343042071	100%	64.13%	10.78%	100%	0.64126	0.938	1.641
42.5816023738872	100%	64.28%	10.82%	100%	0.64275	0.938	1.643
42.6004728132388	100%	64.42%	10.86%	100%	0.64425	0.938	1.644
42.6395939086294	100%	64.57%	10.9%	100%	0.64574	0.938	1.646
42.9963235294118	100%	64.72%	10.94%	100%	0.64723	0.938	1.647
43.3060109289617	100%	64.87%	10.98%	100%	0.64873	0.938	1.649
43.5103244837758	100%	65.02%	11.03%	100%	0.65022	0.938	1.650
43.5227272727273	100%	65.17%	11.07%	100%	0.65172	0.938	1.652
43.7784090909091	100%	65.32%	11.11%	100%	0.65321	0.938	1.653
43.8888888888889	100%	65.47%	11.15%	100%	0.65471	0.938	1.655
44.0810810810811	100%	65.62%	11.2%	100%	0.65620	0.938	1.656
44.1685649202733	100%	65.77%	11.24%	100%	0.65770	0.938	1.658
44.2333333333333	100%	65.92%	11.28%	100%	0.65919	0.938	1.659
44.3204868154158	100%	66.07%	11.33%	100%	0.66069	0.938	1.661
44.3582887700535	100%	66.22%	11.37%	100%	0.66218	0.938	1.662
44.3922651933702	100%	66.37%	11.42%	100%	0.66368	0.938	1.664
44.4496487119438	100%	66.52%	11.46%	100%	0.66517	0.938	1.665
44.5301204819277	100%	66.67%	11.51%	100%	0.66667	0.938	1.667
44.7948717948718	100%	66.82%	11.55%	100%	0.66816	0.938	1.668
44.9101796407186	100%	66.97%	11.6%	100%	0.66966	0.938	1.670
44.9786324786325	100%	67.12%	11.65%	100%	0.67115	0.938	1.671
45.0749063670412	100%	67.26%	11.69%	100%	0.67265	0.938	1.673
45.2091254752852	100%	67.41%	11.74%	100%	0.67414	0.938	1.674
45.4245283018868	100%	67.56%	11.79%	100%	0.67564	0.938	1.676
45.5081967213115	100%	67.71%	11.84%	100%	0.67713	0.938	1.677
45.5298013245033	100%	67.86%	11.89%	100%	0.67862	0.938	1.679
45.5361596009975	100%	68.01%	11.93%	100%	0.68012	0.938	1.680
45.6666666666667	100%	68.16%	11.98%	100%	0.68161	0.938	1.682
45.7110609480813	100%	68.31%	12.03%	100%	0.68311	0.938	1.683
45.9101654846336	100%	68.46%	12.08%	100%	0.68460	0.938	1.685
46.0127931769723	100%	68.61%	12.13%	100%	0.68610	0.938	1.686
46.0436893203883	100%	68.76%	12.18%	100%	0.68759	0.938	1.688
46.3356164383562	100%	68.91%	12.24%	100%	0.68909	0.938	1.689
46.4383561643836	100%	69.06%	12.29%	100%	0.69058	0.938	1.691
47.2853828306264	100%	69.21%	12.34%	100%	0.69208	0.938	1.692
47.3618090452261	100%	69.36%	12.39%	100%	0.69357	0.938	1.694
47.3939393939394	100%	69.51%	12.45%	100%	0.69507	0.938	1.695
48.2198952879581	100%	69.66%	12.5%	100%	0.69656	0.938	1.697
48.3636363636364	100%	69.81%	12.55%	100%	0.69806	0.938	1.698
48.5398230088496	100%	69.96%	12.61%	100%	0.69955	0.938	1.700
48.6073059360731	100%	70.1%	12.66%	100%	0.70105	0.938	1.701
48.6526315789474	100%	70.25%	12.72%	100%	0.70254	0.938	1.703
48.6915887850467	100%	70.4%	12.78%	100%	0.70404	0.938	1.704
48.7031700288184	100%	70.55%	12.83%	100%	0.70553	0.938	1.706
49.5463510848126	100%	70.7%	12.89%	100%	0.70703	0.938	1.707

Scale: D-DİMER/PCO2

49.7959183673469	100%	70.85%	12.95%	100%	0.70852	0.938	1.709
49.9563318777293	100%	71%	13%	100%	0.71001	0.938	1.710
49.9719887955182	100%	71.15%	13.06%	100%	0.71151	0.938	1.712
50.0183486238532	100%	71.3%	13.12%	100%	0.71300	0.938	1.713
50.1285347043702	100%	71.45%	13.18%	100%	0.71450	0.938	1.714
50.1666666666667	100%	71.6%	13.24%	100%	0.71599	0.938	1.716
50.3405572755418	100%	71.75%	13.3%	100%	0.71749	0.938	1.717
50.6732673267327	100%	71.9%	13.36%	100%	0.71898	0.938	1.719
50.6808510638298	100%	72.05%	13.43%	100%	0.72048	0.938	1.720
51.3447432762836	100%	72.2%	13.49%	100%	0.72197	0.938	1.722
51.4512471655329	100%	72.35%	13.55%	100%	0.72347	0.938	1.723
51.6040100250627	100%	72.5%	13.62%	100%	0.72496	0.938	1.725
51.6258351893096	100%	72.65%	13.68%	100%	0.72646	0.938	1.726
51.7538461538462	100%	72.8%	13.74%	100%	0.72795	0.938	1.728
51.9111111111111	100%	72.94%	13.81%	100%	0.72945	0.938	1.729
52.0776255707763	100%	73.09%	13.88%	100%	0.73094	0.938	1.731
52.1487603305785	100%	73.24%	13.94%	100%	0.73244	0.938	1.732
52.6719576719577	100%	73.39%	14.01%	100%	0.73393	0.938	1.734
52.807881773399	100%	73.54%	14.08%	100%	0.73543	0.938	1.735
53.1536388140162	100%	73.69%	14.15%	100%	0.73692	0.938	1.737
53.8533834586466	100%	73.84%	14.22%	100%	0.73842	0.938	1.738
54.8192771084337	100%	73.99%	14.29%	100%	0.73991	0.938	1.740
55.7366771159875	100%	74.14%	14.36%	100%	0.74141	0.938	1.741
56.4724919093851	100%	74.29%	14.43%	100%	0.74290	0.938	1.743
56.8571428571429	100%	74.44%	14.5%	100%	0.74439	0.938	1.744
57.3780487804878	100%	74.59%	14.57%	100%	0.74589	0.938	1.746
57.4821852731591	100%	74.74%	14.65%	100%	0.74738	0.938	1.747
57.7536231884058	100%	74.89%	14.72%	100%	0.74888	0.938	1.749
57.9445727482679	100%	75.04%	14.8%	100%	0.75037	0.938	1.750
57.97783933518	100%	75.19%	14.87%	100%	0.75187	0.938	1.752
58.1303116147309	100%	75.34%	14.95%	100%	0.75336	0.938	1.753
58.1440443213296	100%	75.49%	15.03%	100%	0.75486	0.938	1.755
58.5675675675676	100%	75.64%	15.1%	100%	0.75635	0.938	1.756
58.938679245283	100%	75.78%	15.18%	100%	0.75785	0.938	1.758
59.0818858560794	100%	75.93%	15.26%	100%	0.75934	0.938	1.759
60.027397260274	100%	76.08%	15.34%	100%	0.76084	0.938	1.761
60.1069518716578	100%	76.23%	15.43%	100%	0.76233	0.938	1.762
60.2179176755448	100%	76.38%	15.51%	100%	0.76383	0.938	1.764
60.2640264026403	100%	76.53%	15.59%	100%	0.76532	0.938	1.765
60.4872881355932	100%	76.68%	15.68%	100%	0.76682	0.938	1.767
60.7042253521127	100%	76.83%	15.76%	100%	0.76831	0.938	1.768
61.2518628912072	100%	76.98%	15.85%	100%	0.76981	0.938	1.770
61.275	96.55%	76.98%	15.38%	99.81%	0.73532	0.938	1.735
61.4590747330961	96.55%	77.13%	15.47%	99.81%	0.73682	0.938	1.737
61.7054263565891	96.55%	77.28%	15.56%	99.81%	0.73831	0.938	1.738
61.8895348837209	93.1%	77.28%	15.08%	99.61%	0.70383	0.938	1.704
62.5925925925926	93.1%	77.43%	15.17%	99.62%	0.70532	0.938	1.705
62.7327327327327	93.1%	77.58%	15.25%	99.62%	0.70682	0.938	1.707
63.9400921658986	93.1%	77.73%	15.34%	99.62%	0.70831	0.938	1.708
64.1304347826087	93.1%	77.88%	15.43%	99.62%	0.70981	0.938	1.710
64.4946808510638	93.1%	78.03%	15.52%	99.62%	0.71130	0.938	1.711
65.1980198019802	89.66%	78.03%	15.03%	99.43%	0.67682	0.938	1.677
65.2579034941764	89.66%	78.18%	15.12%	99.43%	0.67832	0.938	1.678

Scale: D-DİMER/PCO2

65.2957746478873	89.66%	78.33%	15.2%	99.43%	0.67981	0.938	1.680
65.5365853658537	89.66%	78.48%	15.29%	99.43%	0.68131	0.938	1.681
65.6010230179028	89.66%	78.62%	15.38%	99.43%	0.68280	0.938	1.683
67.202216066482	89.66%	78.77%	15.48%	99.43%	0.68429	0.938	1.684
67.3913043478261	89.66%	78.92%	15.57%	99.44%	0.68579	0.938	1.686
67.4172185430464	89.66%	79.07%	15.66%	99.44%	0.68728	0.938	1.687
67.9669030732861	89.66%	79.22%	15.76%	99.44%	0.68878	0.938	1.689
68.0769230769231	89.66%	79.37%	15.85%	99.44%	0.69027	0.938	1.690
69.3714285714286	89.66%	79.52%	15.95%	99.44%	0.69177	0.938	1.692
70.3076923076923	89.66%	79.67%	16.05%	99.44%	0.69326	0.938	1.693
70.4129793510324	89.66%	79.82%	16.15%	99.44%	0.69476	0.938	1.695
70.7744107744108	89.66%	79.97%	16.25%	99.44%	0.69625	0.938	1.696
72.4701195219124	89.66%	80.12%	16.35%	99.44%	0.69775	0.938	1.698
73.9572192513369	89.66%	80.27%	16.46%	99.44%	0.69924	0.938	1.699
74.4444444444444	89.66%	80.42%	16.56%	99.45%	0.70074	0.938	1.701
74.9015317286652	89.66%	80.57%	16.67%	99.45%	0.70223	0.938	1.702
75.3658536585366	89.66%	80.72%	16.77%	99.45%	0.70373	0.938	1.704
75.6120527306968	89.66%	80.87%	16.88%	99.45%	0.70522	0.938	1.705
75.8761061946903	89.66%	81.02%	16.99%	99.45%	0.70672	0.938	1.707
77.3493975903614	89.66%	81.17%	17.11%	99.45%	0.70821	0.938	1.708
77.7333333333333	89.66%	81.32%	17.22%	99.45%	0.70971	0.938	1.710
78.2820512820513	89.66%	81.46%	17.33%	99.45%	0.71120	0.938	1.711
78.7971698113208	89.66%	81.61%	17.45%	99.45%	0.71270	0.938	1.713
79.2757660167131	89.66%	81.76%	17.57%	99.45%	0.71419	0.938	1.714
79.572192513369	89.66%	81.91%	17.69%	99.46%	0.71568	0.938	1.716
79.7668997668998	89.66%	82.06%	17.81%	99.46%	0.71718	0.938	1.717
79.7694524495677	89.66%	82.21%	17.93%	99.46%	0.71867	0.938	1.719
80.8067940552017	89.66%	82.36%	18.06%	99.46%	0.72017	0.938	1.720
81.288056206089	89.66%	82.51%	18.18%	99.46%	0.72166	0.938	1.722
81.3165266106442	89.66%	82.66%	18.31%	99.46%	0.72316	0.938	1.723
81.9128329297821	89.66%	82.81%	18.44%	99.46%	0.72465	0.938	1.725
82.0963172804533	86.21%	82.81%	17.86%	99.28%	0.69017	0.938	1.690
83.447619047619	86.21%	82.96%	17.99%	99.28%	0.69167	0.938	1.692
84.4213973799127	82.76%	82.96%	17.39%	99.11%	0.65718	0.938	1.657
84.7912087912088	82.76%	83.11%	17.52%	99.11%	0.65868	0.938	1.659
85.0117647058824	82.76%	83.26%	17.65%	99.11%	0.66017	0.938	1.660
85.5591054313099	82.76%	83.41%	17.78%	99.11%	0.66167	0.938	1.662
85.6132075471698	82.76%	83.56%	17.91%	99.11%	0.66316	0.938	1.663
85.8125	82.76%	83.71%	18.05%	99.12%	0.66466	0.938	1.665
86.1463414634146	82.76%	83.86%	18.18%	99.12%	0.66615	0.938	1.666
86.9373549883991	82.76%	84.01%	18.32%	99.12%	0.66765	0.938	1.668
87.89592760181	82.76%	84.16%	18.46%	99.12%	0.66914	0.938	1.669
88.6849315068493	82.76%	84.3%	18.6%	99.12%	0.67064	0.938	1.671
90.2605210420842	82.76%	84.45%	18.75%	99.12%	0.67213	0.938	1.672
90.4471544715447	82.76%	84.6%	18.9%	99.12%	0.67363	0.938	1.674
90.9862385321101	82.76%	84.75%	19.05%	99.13%	0.67512	0.938	1.675
91.6032608695652	82.76%	84.9%	19.2%	99.13%	0.67661	0.938	1.677
91.9064748201439	82.76%	85.05%	19.35%	99.13%	0.67811	0.938	1.678
92.2222222222222	82.76%	85.2%	19.51%	99.13%	0.67960	0.938	1.680
92.7472527472527	82.76%	85.35%	19.67%	99.13%	0.68110	0.938	1.681
93.1034482758621	82.76%	85.5%	19.83%	99.13%	0.68259	0.938	1.683
93.8055555555556	82.76%	85.65%	20%	99.13%	0.68409	0.938	1.684
93.9364303178484	82.76%	85.8%	20.17%	99.14%	0.68558	0.938	1.686

Scale: D-DİMER/PCO2

94.7322970639033	82.76%	85.95%	20.34%	99.14%	0.68708	0.938	1.687
94.7552447552447	82.76%	86.1%	20.51%	99.14%	0.68857	0.938	1.689
96.5194805194805	79.31%	86.1%	19.83%	98.97%	0.65409	0.938	1.654
96.5324384787472	79.31%	86.25%	20%	98.97%	0.65558	0.938	1.656
96.7605633802817	79.31%	86.4%	20.18%	98.97%	0.65708	0.938	1.657
98.8712522045855	79.31%	86.55%	20.35%	98.97%	0.65857	0.938	1.659
99.5907928388747	79.31%	86.7%	20.54%	98.98%	0.66007	0.938	1.660
100.356294536817	79.31%	86.85%	20.72%	98.98%	0.66156	0.938	1.662
101.525423728814	79.31%	87%	20.91%	98.98%	0.66306	0.938	1.663
101.526717557252	79.31%	87.14%	21.1%	98.98%	0.66455	0.938	1.665
102.463768115942	79.31%	87.29%	21.3%	98.98%	0.66605	0.938	1.666
103.070707070707	79.31%	87.44%	21.5%	98.98%	0.66754	0.938	1.668
104.790286975717	79.31%	87.59%	21.7%	98.99%	0.66904	0.938	1.669
105.911330049261	79.31%	87.74%	21.9%	98.99%	0.67053	0.938	1.671
106.88829787234	79.31%	87.89%	22.12%	98.99%	0.67203	0.938	1.672
108.141135972461	79.31%	88.04%	22.33%	98.99%	0.67352	0.938	1.674
110.531914893617	79.31%	88.19%	22.55%	98.99%	0.67502	0.938	1.675
113.324468085106	79.31%	88.34%	22.77%	98.99%	0.67651	0.938	1.677
113.353293413174	79.31%	88.49%	23%	99%	0.67801	0.938	1.678
113.895705521472	79.31%	88.64%	23.23%	99%	0.67950	0.938	1.680
114.093264248705	79.31%	88.79%	23.47%	99%	0.68100	0.938	1.681
114.425287356322	79.31%	88.94%	23.71%	99%	0.68249	0.938	1.682
115.447368421053	79.31%	89.09%	23.96%	99%	0.68399	0.938	1.684
115.851648351648	79.31%	89.24%	24.21%	99%	0.68548	0.938	1.685
116.619318181818	79.31%	89.39%	24.47%	99.01%	0.68697	0.938	1.687
117.251908396947	79.31%	89.54%	24.73%	99.01%	0.68847	0.938	1.688
119.700460829493	79.31%	89.69%	25%	99.01%	0.68996	0.938	1.690
122.613065326633	79.31%	89.84%	25.27%	99.01%	0.69146	0.938	1.691
123.615635179153	79.31%	89.99%	25.56%	99.01%	0.69295	0.938	1.693
124.349112426036	79.31%	90.13%	25.84%	99.01%	0.69445	0.938	1.694
125.961123110151	79.31%	90.28%	26.14%	99.02%	0.69594	0.938	1.696
128.903743315508	79.31%	90.43%	26.44%	99.02%	0.69744	0.938	1.697
129.076433121019	79.31%	90.58%	26.74%	99.02%	0.69893	0.938	1.699
130.049751243781	79.31%	90.73%	27.06%	99.02%	0.70043	0.938	1.700
131.211267605634	75.86%	90.73%	26.19%	98.86%	0.66595	0.938	1.666
132.196531791908	75.86%	90.88%	26.51%	98.86%	0.66744	0.938	1.667
132.682403433476	75.86%	91.03%	26.83%	98.86%	0.66893	0.938	1.669
133.302961275626	75.86%	91.18%	27.16%	98.87%	0.67043	0.938	1.670
134.168797953964	75.86%	91.33%	27.5%	98.87%	0.67192	0.938	1.672
134.313099041534	75.86%	91.48%	27.85%	98.87%	0.67342	0.938	1.673
137.492537313433	75.86%	91.63%	28.21%	98.87%	0.67491	0.938	1.675
139.121447028424	75.86%	91.78%	28.57%	98.87%	0.67641	0.938	1.676
140.455486542443	75.86%	91.93%	28.95%	98.87%	0.67790	0.938	1.678
140.758928571429	75.86%	92.08%	29.33%	98.88%	0.67940	0.938	1.679
141.78674351585	72.41%	92.08%	28.38%	98.72%	0.64492	0.938	1.645
145.1197053407	68.97%	92.08%	27.4%	98.56%	0.61043	0.938	1.610
147.328244274809	68.97%	92.23%	27.78%	98.56%	0.61193	0.938	1.612
147.507886435331	68.97%	92.38%	28.17%	98.56%	0.61342	0.938	1.613
147.521865889213	68.97%	92.53%	28.57%	98.57%	0.61492	0.938	1.615
149.94623655914	68.97%	92.68%	28.99%	98.57%	0.61641	0.938	1.616
150.182291666667	68.97%	92.83%	29.41%	98.57%	0.61791	0.938	1.618
150.371352785146	65.52%	92.83%	28.36%	98.42%	0.58342	0.938	1.583
152.307692307692	65.52%	92.97%	28.79%	98.42%	0.58492	0.938	1.585

Scale: D-DİMER/PCO2

156.06648199446	65.52%	93.12%	29.23%	98.42%	0.58641	0.938	1.586
158.974358974359	65.52%	93.27%	29.69%	98.42%	0.58791	0.938	1.588
161.349693251534	65.52%	93.42%	30.16%	98.43%	0.58940	0.938	1.589
161.743869209809	65.52%	93.57%	30.65%	98.43%	0.59090	0.938	1.591
162.409638554217	65.52%	93.72%	31.15%	98.43%	0.59239	0.938	1.592
164.642857142857	65.52%	93.87%	31.67%	98.43%	0.59389	0.938	1.594
165.454545454545	65.52%	94.02%	32.2%	98.44%	0.59538	0.938	1.595
165.46485260771	65.52%	94.17%	32.76%	98.44%	0.59688	0.938	1.597
166.478873239437	62.07%	94.17%	31.58%	98.28%	0.56239	0.938	1.562
168.399071925754	58.62%	94.17%	30.36%	98.13%	0.52791	0.938	1.528
169.707446808511	58.62%	94.32%	30.91%	98.13%	0.52941	0.938	1.529
171.701149425287	58.62%	94.47%	31.48%	98.14%	0.53090	0.938	1.531
173.032967032967	58.62%	94.62%	32.08%	98.14%	0.53240	0.938	1.532
174.148351648352	58.62%	94.77%	32.69%	98.14%	0.53389	0.938	1.534
182.527173913043	58.62%	94.92%	33.33%	98.15%	0.53538	0.938	1.535
183.574879227053	58.62%	95.07%	34%	98.15%	0.53688	0.938	1.537
191.800486618005	58.62%	95.22%	34.69%	98.15%	0.53837	0.938	1.538
193.341346153846	58.62%	95.37%	35.42%	98.15%	0.53987	0.938	1.540
195.291005291005	55.17%	95.37%	34.04%	98%	0.50539	0.938	1.505
195.606694560669	55.17%	95.52%	34.78%	98.01%	0.50688	0.938	1.507
199.212827988338	55.17%	95.67%	35.56%	98.01%	0.50838	0.938	1.508
201.845386533666	55.17%	95.81%	36.36%	98.01%	0.50987	0.938	1.510
201.990171990172	55.17%	95.96%	37.21%	98.02%	0.51137	0.938	1.511
204.136690647482	55.17%	96.11%	38.1%	98.02%	0.51286	0.938	1.513
205.045317220544	55.17%	96.26%	39.02%	98.02%	0.51435	0.938	1.514
209.942028985507	55.17%	96.41%	40%	98.02%	0.51585	0.938	1.516
210.33950617284	55.17%	96.56%	41.03%	98.03%	0.51734	0.938	1.517
215.955334987593	55.17%	96.71%	42.11%	98.03%	0.51884	0.938	1.519
219.812030075188	55.17%	96.86%	43.24%	98.03%	0.52033	0.938	1.520
220.05	55.17%	97.01%	44.44%	98.04%	0.52183	0.938	1.522
221.967213114754	55.17%	97.16%	45.71%	98.04%	0.52332	0.938	1.523
224.493243243243	55.17%	97.31%	47.06%	98.04%	0.52482	0.938	1.525
226.643598615917	55.17%	97.46%	48.48%	98.05%	0.52631	0.938	1.526
230.58282208589	55.17%	97.61%	50%	98.05%	0.52781	0.938	1.528
234.134275618375	51.72%	97.61%	48.39%	97.9%	0.49333	0.938	1.493
235.514705882353	51.72%	97.76%	50%	97.9%	0.49482	0.938	1.495
235.688073394495	51.72%	97.91%	51.72%	97.91%	0.49631	0.938	1.496
236.875	51.72%	98.06%	53.57%	97.91%	0.49781	0.938	1.498
258.436578171091	51.72%	98.21%	55.56%	97.91%	0.49930	0.938	1.499
262.27	51.72%	98.36%	57.69%	97.92%	0.50080	0.938	1.501
265.265017667845	48.28%	98.36%	56%	97.77%	0.46632	0.938	1.466
291.596330275229	48.28%	98.51%	58.33%	97.77%	0.46781	0.938	1.468
329.133663366337	48.28%	98.65%	60.87%	97.78%	0.46931	0.938	1.469
376.871035940803	44.83%	98.65%	59.09%	97.63%	0.43482	0.938	1.435
402.204610951009	41.38%	98.65%	57.14%	97.49%	0.40034	0.938	1.400
409.673024523161	37.93%	98.65%	55%	97.35%	0.36586	0.938	1.366
419.906103286385	37.93%	98.8%	57.89%	97.35%	0.36735	0.938	1.367
430.063965884861	37.93%	98.95%	61.11%	97.35%	0.36885	0.938	1.369
455.889724310777	34.48%	98.95%	58.82%	97.21%	0.33436	0.938	1.334
464.649859943978	34.48%	99.1%	62.5%	97.21%	0.33586	0.938	1.336
464.742268041237	31.03%	99.1%	60%	97.07%	0.30138	0.938	1.301
490.695187165775	27.59%	99.1%	57.14%	96.93%	0.26689	0.938	1.267
495.496828752643	24.14%	99.1%	53.85%	96.79%	0.23241	0.938	1.232

Scale: D-DİMER/PCO2

522.68221574344	24.14%	99.25%	58.33%	96.79%	0.23391	0.938	1.234
576.626016260163	20.69%	99.25%	54.55%	96.65%	0.19942	0.938	1.199
577.643312101911	17.24%	99.25%	50%	96.51%	0.16494	0.938	1.165
672.87037037037	13.79%	99.25%	44.44%	96.37%	0.13046	0.938	1.130
699.966555183947	10.34%	99.25%	37.5%	96.23%	0.09597	0.938	1.096
719.651162790698	10.34%	99.4%	42.86%	96.24%	0.09747	0.938	1.097
805.448504983389	6.9%	99.4%	33.33%	96.1%	0.06299	0.938	1.063
864.97461928934	6.9%	99.55%	40%	96.1%	0.06448	0.938	1.064
938.219696969697	3.45%	99.55%	25%	95.97%	0.03000	0.938	1.030
1149.74564926372	0%	99.55%	0%	95.83%	-0.00448	0.938	0.996
1240.36303630363	0%	99.7%	0%	95.83%	-0.00299	0.938	0.997
2615.46712802768	0%	99.85%	0%	95.84%	-0.00149	0.938	0.999
