# Supplementary material for: Pre-CT risk stratification using the D-dimer/pCO₂ ratio in D-dimer–positive emergency department patients: diagnostic accuracy study
Source: BMC Emerg Med. 2025 Nov 17;25:237. doi: 10.1186/s12873-025-01395-6 (PMC12625727; doi:10.1186/s12873-025-01395-6)
Supplement: Supplementary file 3 — Supplementary Material 3 [file 12873_2025_1395_MOESM3_ESM.docx]

| Model | AUC | 95% CI Lower | 95% CI Upper |
| --- | --- | --- | --- |
| D-dimer | 0.809 | 0.770 | 0.844 |
| Age-adjusted D-dimer (continuous) | 0.799 | 0.760 | 0.836 |
| D-dimer/pCO2 ratio | 0.811 | 0.775 | 0.847 |

**Supplementary table 1.** AUC (95% CI) — Bootstrap

AUC values estimated by ROC analysis with 2000 bootstrap resamples. CI = percentile bootstrap 95% confidence interval.
