# Supplementary material for: Pre-CT risk stratification using the D-dimer/pCO₂ ratio in D-dimer–positive emergency department patients: diagnostic accuracy study
Source: BMC Emerg Med. 2025 Nov 17;25:237. doi: 10.1186/s12873-025-01395-6 (PMC12625727; doi:10.1186/s12873-025-01395-6)
Supplement: Supplementary file 4 — Supplementary Material 4 [file 12873_2025_1395_MOESM4_ESM.docx]

| Comparison | ΔAUC | 95% CI Lower | 95% CI Upper | Bootstrap p-value |
| --- | --- | --- | --- | --- |
| Ratio vs D-dimer | 0.002 | -0.018 | 0.022 | 0.620 |
| Ratio vs Age-adjusted D-dimer | 0.012 | -0.007 | 0.031 | 0.210 |
| D-dimer vs Age-adjusted D-dimer | 0.010 | -0.010 | 0.030 | 0.330 |

**Supplementary table 2.** AUC Comparisons (ΔAUC, 95% CI, bootstrap p)

ΔAUC values represent mean differences from bootstrap resamples. p-values estimated as two-sided bootstrap proportions.
